# Abscisic Acid-Mediated Drought Tolerance in *Populus*: Physiological, Molecular, and Biotechnological Perspectives

**DOI:** 10.3390/cimb48090946

**Published:** 2026-09-16

**Authors:** Sajid Ali, Shakir Ullah, Mansoor Hayat, Hasham Ahmad, Zainab Shahbaz, Muhammad Tariq Khan

**Affiliations:** 1 State Key Laboratory of Tree Genetics and Breeding, Northeast Forestry University, Hexing Road, Harbin 150040, China; sajidaliaup@gmail.com; 2 College of Chemistry, Chemical Engineering and Resource Utilization, Northeast Forestry University, Harbin 150040, China; 3 College of Food and Health, Northeast Forestry University, Harbin 150040, China; 4 School of Forestry, Northeast Forestry University, Hexing Road, Harbin 150040, China; mansoor@nefu.edu.cn (M.H.); hasham@nefu.edu.cn (H.A.); zainab103@nefu.edu.cn (Z.S.); 5 Department of Science and Environmental Studies, The Education University of Hong Kong, Tai Po, New Territories, Hong Kong 999077, China

**Keywords:** abscisic acid, *Populus*, drought tolerance, ABA signaling, multi-omics, epigenetic regulation

## Abstract

Drought stress is a major constraint on global forestry productivity and poses a significant threat to economically important tree species such as *Populus*. Abscisic acid (ABA) signaling plays a central role in drought tolerance by regulating molecular, physiological, and biochemical processes. This review summarizes current knowledge of ABA signaling pathways and their roles in drought resilience in *Populus*. It covers physiological responses, molecular regulatory networks, hormonal crosstalk, biotechnological advances, and emerging field applications. Core ABA signaling components, including *PYR*/*PYL*/*RCAR* receptors, *PP2Cs*, *SnRK2* kinases, and transcription factors such as *ABFs*, *NACs*, and *MYBs*, coordinate adaptive responses involved in stomatal regulation, osmotic adjustment, and root system remodeling. Integrative omics approaches, like transcriptomics, proteomics, metabolomics, and epigenomics, have provided mechanistic insights into ABA-mediated drought responses, although some regulatory mechanisms are supported by conserved evidence from model plants and require further validation in *Populus*. Recent biotechnological advances, including gene overexpression, RNA interference (RNAi), CRISPR/Cas9 genome editing, and emerging CRISPR-based regulatory approaches, provide new opportunities for manipulating ABA-associated drought responses in *Populus*. While several genome-editing and genetic approaches have been experimentally validated in *Populus*, many ABA-related targets remain prospective strategies requiring further functional evaluation. However, practical implementation remains limited by an incomplete understanding of hormonal crosstalk, insufficient characterization of epigenetic regulation, and ecological and social concerns. Addressing these challenges requires ecological trials, deeper analyses of hormone networks, advanced epigenetic research, robust environmental risk assessments, and interdisciplinary integration. Future progress will depend on linking molecular discoveries with physiological validation and long-term field evaluation to develop drought-resilient *Populus* cultivars for sustainable forestry under changing climate conditions.

## 1. Introduction

Drought stress is one of the primary abiotic factors impacting forest ecosystems globally and has significant impacts on tree growth, biomass accumulation, physiological responses, and longevity [1]. Droughts are more frequent and more intense under current climate change, posing an increasing threat to forest productivity, ecosystem stability, and carbon sequestration. According to assessments, drought frequency and intensity have increased globally in recent decades, but the degree of the rise varies by geographic region, drought definition, and method of evaluation [2]. Although significant advances have been made in the field of drought response in herbaceous model plants, the molecular and physiological mechanisms underlying long-term drought adaptation in woody perennials such as *Populus* remain incompletely understood. *Populus* is an important forest tree species and has been adopted as a model system in the study of drought responses among forest tree genera due to its rapid growth, high transformation efficiency, well-annotated genome, and economic value [3]. *Populus* is a widely used species in plantations around the world, mainly in temperate areas such as China, Europe, and North America, and is an important species in commercial forestry and agroforestry systems [4]. Under drought conditions, *Populus* exhibits reduced photosynthetic capacity, reduced gas exchange, altered plant water relations, and decreased growth, leading to reduced carbon assimilation and biomass production. Such physiological changes have been linked to hormonal signaling pathway changes, especially the abscisic acid (ABA) signaling network, which orchestrates several drought-acclimation responses [5,6].

A major phytohormone responsible for plant responses to water deficit, ABA plays a major role in stomatal closure, which helps to maintain plant water balance [7,8]. When a drought is sensed, ABA levels rise in plants’ roots and leaves, leading to a signaling pathway involving *PYR*/*PYL*/*RCAR* receptors, protein phosphatase 2Cs (*PP2Cs*) and SNF1-related protein kinases 2 (*SnRK2s*) [9,10]. These core components regulate downstream transcription factors and ABA-responsive genes involved in drought adaptation. Several ABA-related genes such as *NCED*-, *PYL*- and *SnRK2*-related components of stress signaling and drought-responsive physiological regulation are responsive to drought stress in *Populus* [7,11]. Ali et al. [12] recently reviewed the general functions of phytohormones, such as ABA in drought responses of *Populus*, and their interaction with other hormonal and root-related activities. The expanding knowledge of ABA homeostasis, perception, signal transduction, and downstream regulatory networks, however, highlights the need for a dedicated synthesis of ABA-mediated drought responses. In this review, the drought adaptation strategies of ABA will therefore be explored in *Populus*, focusing on physiological regulation, ABA signaling pathways, transcriptional regulation, hormonal cross-talk, multi-omics and epigenetic regulation, and emerging biotechnological approaches. It also highlights existing gaps in field validation, genotype-specific responses, and the use of genome-editing technologies in woody forest species.

## 2. ABA Signaling and Physiological Responses to Drought Stress in *Populus*

**Figure 1:**
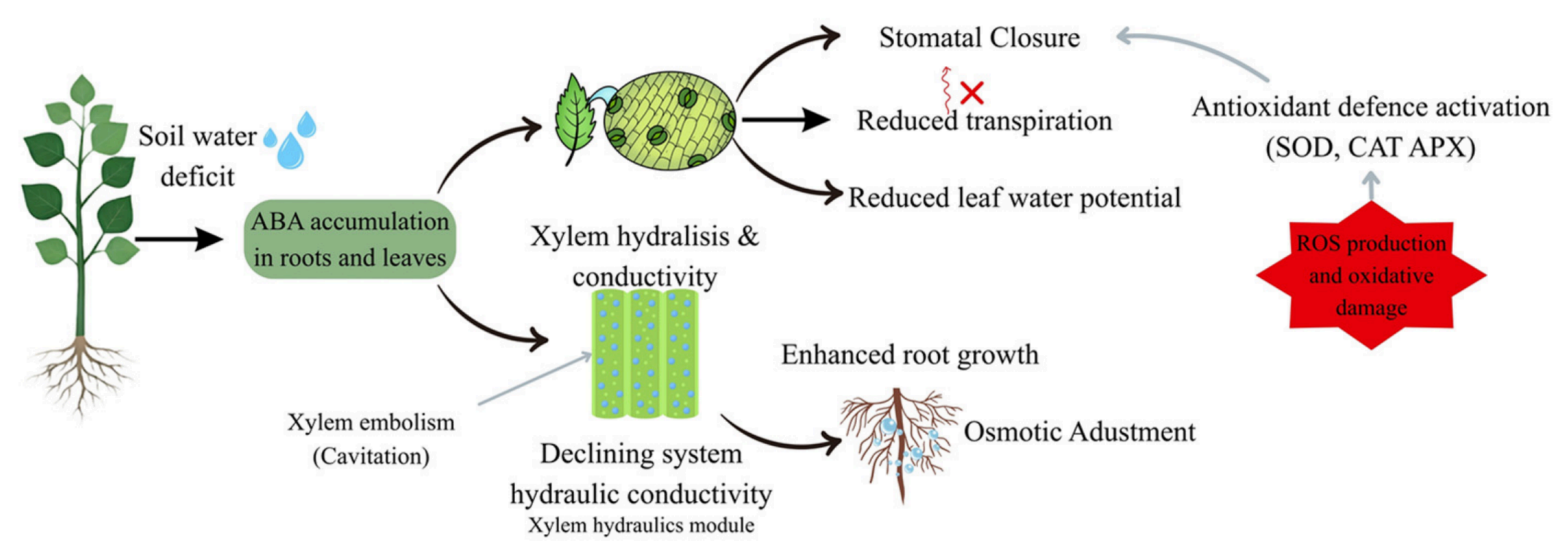
Major physiological and biochemical responses of *Populus* to drought stress, including ABA accumulation, reduced transpiration and leaf water potential, osmotic adjustment, changes in root growth, and ROS-associated oxidative responses. Created in https://BioRender.com (accessed on 8 June 2026).

When drought stress occurs, it causes imbalances in the water homeostasis of the plant and triggers a series of related physiological and biochemical changes that affect growth, physiological function, and survival [13] (Figure 1). In *Populus*, drought responses are highly variable among species and genotypes of a species, depending on the genetic background, developmental stage, drought intensity, and other environmental factors [14]. Water limitation impacts a number of closely related processes, such as stomatal regulation, photosynthetic carbon assimilation, hydraulic transport, osmotic adjustment, oxidative balance and biomass allocation [15]. Notably, ABA is a major regulator of many of these responses, including stomatal, hydraulic, photosynthetic and transcriptional changes in water deficit [16,17].

Hence, understanding the physiological responses of *Populus* to drought is important for identifying traits and mechanisms that can be used to improve drought resistance and implement future breeding and biotechnological strategies. Responses to drought stress vary considerably among *Populus* species and between genotypes of the same species, depending on the developmental stage, drought intensity and environment [18,19]. Stomatal conductance, CO_2_ assimilation, and chloroplast function are usually reduced by drought stress, and thus the photosynthetic efficiency is lowered. Drought stress in *Populus* also has a significant impact on photosynthetic functioning through both alterations in chloroplast function and gas exchange [20,21]. Stomatal closure mediated by ABA is one of the important water conservation responses in *Populus* but more persistent decreases in stomatal conductance also decrease carbon assimilation and growth, which is a compromise between water conservation and biomass production. In poplar, several ABA-responsive signaling components and transcriptional regulators have been proven to participate in the ABA-dependent stomatal behavior regulation [22]. However, drought-induced changes in carbon assimilation can also alter biomass allocation, although the magnitude and direction of this response depend on genotype and stress conditions.

Drought also strongly affects the hydraulic functioning of *Populus*. Under water deficit, the more negative the xylem water potential, the greater the risk of cavitation, leading to embolism formation and a gradual reduction in xylem hydraulic conductivity [23,24]. Genotypic variation in stem cavitation resistance in *P. nigra* was found to be significant by Guet et al. [25], with xylem tension causing 50% loss of hydraulic conductivity (Ψ50) ranging from about −1.72 to −2.31 MPa for 33 genotypes, and the average being −2.01 MPa. These results illustrate the high degree of hydraulic vulnerability within *Populus*, which cannot be explained solely by vessel diameter. Hydraulic impairment during drought may result in a decrease in water transport to leaves, stomatal closure, loss of turgor, and ultimately limitation of canopy function [26]. Although the mechanisms of drought-induced hydraulic failure are increasingly well understood, the ability of embolized xylem in established *Populus* trees to recover after multiple drought events is still poorly resolved [27]. In particular, at the organ level, the progressive hydraulic dysfunction may spread from the stem xylem to the vascular system of the leaves, where the connectivity of the conduits and the hierarchy of the venation network may affect the spread and onset of embolism [28]. Consequently, embolism is not evenly distributed across leaves. Rather, variation in vulnerability between vein orders can lead to a spatially heterogeneous pattern of hydraulic failure, where larger veins can serve as preferential nucleation points for embolism and then act as pathways for its spread through the vascular network [29]. A strong gradient of dehydration susceptibility has been observed and is related to the hierarchical level of veins: the susceptibility to embolism increases with vein size, with the largest veins being the first to embolize [30]. As leaf water content decreases due to hydraulic disruption, the less water is available to the leaf tissues, leading to wilting, senescence and a decrease in leaf area and canopy development [31]. However, in response, leaves rapidly close their stomata to modulate stomatal conductance (gs) and lower the process of transpiration [24,32]. ABA activates *SnRK2* kinases through binding to *PYR*/*PYL* receptors and inhibiting *PP2C* phosphatases, which in turn leads to stomatal closure and a decrease in transpiration, limiting further water loss. However, reduced transpiration further limits evaporative cooling of the leaf surface, which can increase leaf temperature and intensify thermal and photooxidative stress, resulting in reactive oxygen species (ROS) production and oxidative damage.

Furthermore, osmotic adjustment may involve the accumulation of compatible solutes such as proline, sugars, glycine betaine and inorganic ions that play a role in maintaining membrane integrity and cellular function under dehydration stress [33]. Changes in soluble sugars, proline, amino acids, and other osmotically active metabolites as a result of drought have been studied both within and between *Populus* species and hybrids, and the relative contribution of the various solutes varies depending on both species/genotypes and drought intensity [34,35]. The osmotic potential and ability to osmotically adjust are also found to be significantly different between *Populus* clones in field and controlled environment studies [36,37]. So, osmotic adjustment is not necessarily the same as a fixed increase in relative water content, but rather a way to sustain turgor and metabolic activities at decreasing water potential, which varies among genotypes, environments, and drought severity. Furthermore, drought stress also causes changes in the redox status in the cells of *Populus* and can enhance the accumulation of ROS, such as H_2_O_2_ and superoxide (O_2_^−^), and excessive ROS accumulation can lead to oxidative damage [38,39]. In order to reduce the accumulation of ROS and keep the redox status of the cells, *Populus* activates a group of antioxidant enzymes, including superoxide dismutase (SOD), catalase (CAT), and ascorbate peroxidase (APX) [40,41]. Specifically, in *P. euphratica*, drought tolerance is enhanced by drought-induced ABA accumulation in plant tissues, which promotes H_2_O_2_ production and ABA-induced stomatal closure, thereby showing that controlled ROS production can positively affect ABA-mediated drought stress alleviation [42]. Thus, drought resistance in *Populus* is achieved through a combination of hydraulic regulation, osmotic adjustment, ABA signaling, redox homeostasis, and growth allocation [34,37] (Table 1). Further research is needed to more accurately forecast drought resilience and productivity in *Populus* plantations under future climate conditions, incorporating physiological, molecular, and long-term field analyses.

**Table 1:** Major drought-associated physiological and biochemical responses in *Populus* species and their reported links with ABA-mediated drought adaptation.

Physiological and Biochemical Attributes	*Populus* Species/Genotype	Drought Response in *Populus*	ABA Mode of Action	References
Stomatal conductance	*P. nigra*; hybrid poplar clones	Stomatal conductance (gs) declines as water stress intensifies, restricting water loss.	In *P. nigra*, drought-induced changes in ABA biosynthesis and accumulation are closely coordinated with stomatal, mesophyll, and hydraulic conductance. PeABF3 overexpression also increases ABA sensitivity and promotes stomatal closure in poplar.	[15]
Photosynthetic rate	Hybrid clones DN-34, R-247 and OP-367; *P. tomentosa* transgenic lines	Net photosynthetic rate decreases with increasing drought severity, although the magnitude is genotype dependent.	*PeABF3*-overexpressing poplars maintained higher photosynthetic activity and WUE during drought through enhanced ABA-responsive stomatal regulation.	[35,43]
Root morphology and allocation	*P. euphratica*	Drought decreases absolute root depth but increases relative root depth through changes in root architecture and greater relative allocation to the root/taproot system.	A direct ABA mechanism was not tested in this root-phenotyping study; therefore, an ABA-specific mechanism should not be assigned to this response.	[44]
ROS and antioxidant defense	*P. kangdingensis*, *P. cathayana*; transgenic 84K poplar	Drought increases H_2_O_2_ and MDA, accompanied by changes/increases in CAT, SOD, POD, APX and GR activities.	*PeCHYR1* links ABA signaling with controlled H_2_O_2_ production in guard cells, promoting stomatal closure and drought tolerance in transgenic poplar.	[41,42]
Leaf water status/potential	*P. nigra*; *P. × canescens*	Leaf or predawn water potential becomes more negative as soil water availability declines.	In *P. nigra*, drought strongly increases ABA accumulation and *NCED3* expression, accompanying coordinated hydraulic and gas-exchange adjustments.	[15,45]
Chlorophyll fluorescence/PSII function	Hybrid clones Nanlin 1388 and Nanlin 895; *PeABF3*-overexpressing *P. tomentosa*	Prolonged water stress decreases photochemical quenching (qL) and effective PSII quantum yield [Y(II)], while NPQ generally shows an opposite response.	PeABF3-overexpressing poplar maintained more stable Fv/Fm and photosynthetic activity under drought, together with enhanced ABA sensitivity.	[43,46]
Transpiration rate	Hybrid clones DN-34, R-247 and OP-367; *P. × canescens*	Transpiration decreases significantly during drought, generally in parallel with declining stomatal conductance.	ABA-associated stomatal regulation provides a direct mechanism for reducing water loss in *Populus*, as demonstrated in *P. nigra* and PeABF3 transgenic poplar.	[15,35,43]
Growth and biomass accumulation	*PsDUF6A*-overexpressing 84K poplar	Under drought, transgenic 84K poplar maintained greater root biomass than non-transgenic 84K poplar.	PsDUF6A overexpression enhanced drought tolerance by improving root biomass, photosynthetic activity, reducing water loss, and promoting antioxidant activity and ROS scavenging.	[47]

## 3. ABA Biosynthesis, Transport, and Systemic Signaling Networks in *Populus*

**Figure 2:**
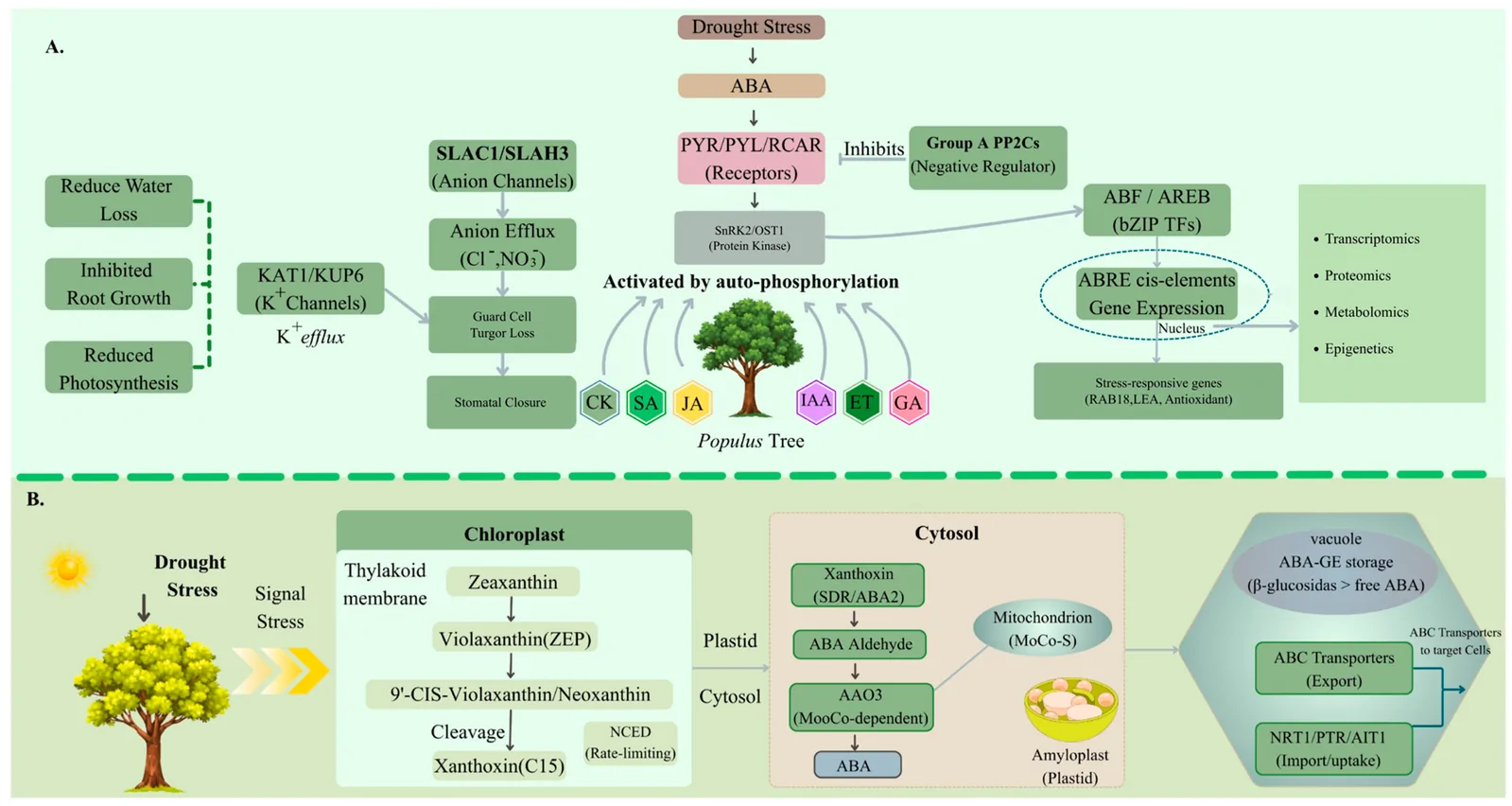
Overview of ABA signaling, biosynthesis, and transport pathways in *Populus* during drought stress. (**A**) Core ABA signaling pathway showing ABA perception by *PYR/PYL/RCAR* receptors, inhibition of PP2C phosphatases, activation of *SnRK2* kinases, and regulation of downstream transcription factors, ion channels, and stress-responsive genes. (**B**) ABA biosynthesis and transport pathway, illustrating carotenoid-derived ABA production, cytosolic conversion steps, and ABA storage, export, and uptake mechanisms involved in drought adaptation. Solid arrows indicate direct metabolic pathway flow or activation, blunt-ended lines represent inhibitory interactions, and circles and rectangular boxes denote cellular compartments, hormones, components, and physiological response nodes. Created in https://BioRender.com (accessed on 8 June 2026).

Understanding the mechanisms of ABA production and transport is important for comprehending its involvement in drought tolerance. In plants, abscisic acid (ABA) serves as a principal regulatory molecule that modulates responses to several abiotic stressors, including drought, salinity, and cold [48,49]. Specifically, in *Populus*, the ABA production and transport pathways are rapidly activated in response to drought cues, leading to systemic stress-responsive physiological and molecular acclimations (Figure 2A,B). The biosynthesis of ABA is mainly conducted in the chloroplast via the carotenoid pathway: the carotenoid precursors for the biosynthesis of ABA (violaxanthin and neoxanthin) are converted to ABA intermediates [50]. The oxidative cleavage of 9-cis-epoxycarotenoids, primarily 9-cis-violaxanthin and 9′-cis-neoxanthin, by 9-cis-epoxycarotenoid dioxygenase (*NCED*) constitutes the first committed and rate-limiting step of ABA biosynthesis. The cleavage product, xanthoxin, is then metabolized to abscisic aldehyde in the cytosol by a short-chain alcohol dehydrogenase/reductase (ABA2/SDR). The final step is the oxidation of abscisic aldehyde to ABA by abscisic aldehyde oxidase (AAO) which is the last enzyme in the biosynthetic pathway [51,52].

*NCED* genes occur as multigene families in many plant species although individual family members may differ in their expression and stress responsiveness. The induction of *NCED* genes under drought stress is fairly quick as a significant increase in the *NCED* transcript accumulation has been observed within 15 to 30 min of dehydration treatment or leaf detachment [53,54]. Similarly, transcriptomic and qRT-PCR analysis in *P. davidiana* revealed that the expression patterns of the two drought-responsive *NCED* genes, *PdNCED1* and *PdNCED3*, were elevated by more than 5- and 7-fold, respectively, in drought-tolerant cultivars following a short dehydration treatment, but downregulated in the drought-sensitive plants by 2- and 2.9-fold, respectively. This contrasting transcriptional response was also associated with higher ABA accumulation in the drought-tolerant genotypes, demonstrating the importance of the ABA biosynthesis in genotype-specific drought responses [7]. Transcriptomic analysis of *P. davidiana* further revealed considerable time-dependent changes in the expression of drought-responsive genes following 6 and 12 h of polyethylene glycol (PEG)-induced water-deficit treatment [55]. These findings highlight the temporal dynamics of drought-responsive gene regulation in *P. davidiana* and complement the genotype-specific *NCED* responses described above. However, most available evidence has been obtained under controlled laboratory or greenhouse conditions, and the stability and ecological relevance of these ABA-mediated responses under complex field conditions remain insufficiently characterized.

Consistent with these findings, increased ABA accumulation is associated with stomatal regulation, reduced water loss, and improved water-use efficiency, thereby contributing to drought tolerance [56]. At the subcellular level, compartmentalization of ABA biosynthesis provides additional regulatory control over the pathway. *NCED* enzymes are localized in the chloroplast, where they regulate carotenoid cleavage as a key step in drought-induced ABA production [57]. However, SDR and abscisic aldehyde oxidase (AAO) enzymes are localized in the cytosol, necessitating an efficient pathway for the movement of intermediates, such as xanthoxin from the chloroplast to the cytosol [58] (Table 2). This spatial separation of successive biosynthetic steps provides an additional level of regulation over ABA production and accumulation, enabling coordinated spatial and temporal responses to drought. However, the mechanisms coordinating chloroplast and cytosol-localized ABA biosynthetic steps under changing environmental conditions remain insufficiently understood.

**Table 2:** Key components of ABA biosynthesis and transport relevant to drought responses in *Populus*: established functions and current evidence status.

Component	Function	Location/Transport Route	Evidence Relevant to *Populus*	Evidence Status	References
*NCED* (*PdNCED1*, *PdNCED3*)	Cleaves 9-cis-epoxycarotenoids to form xanthoxin, a committed step in ABA biosynthesis	Chloroplasts/plastid	*PdNCED1* and *PdNCED3* are drought-responsive in *P. davidiana*; manipulation of *PdNCED3* alters ABA accumulation and dehydration tolerance	Direct functional evidence in poplar; plastid localization of *NCED* established in model plants	[7,59]
ABA2/SDR	Converts xanthoxin to abscisic aldehyde	Cytosol	The biochemical function is well established in *Arabidopsis*, but equivalent functional characterization has not been demonstrated in *Populus* in the cited literature	Established in model plants; not functionally validated in *Populus*	[60]
AAO3	Oxidizes abscisic aldehyde to ABA in the final step of ABA biosynthesis	Cytosol	AAO3 function and cytosolic localization are established in *Arabidopsis*; direct functional evidence for an equivalent ABA-biosynthetic AAO3 in *Populus* remains limited	Established in model plants; not functionally validated in *Populus*	[61,62]
AtABCG25	ATP-dependent ABA exporter	Plasma membrane	ABCG-family homologs occur in *Populus*, but ABA-export activity equivalent to AtABCG25 has not been functionally demonstrated in *Populus*	ABA transport demonstrated in *Arabidopsis*, not *Populus*	[63,64]
AtABCG40	Mediates cellular ABA uptake; contributes to ABA-dependent stomatal regulation	Plasma membrane	No equivalent ABCG40-mediated ABA uptake function has yet been functionally demonstrated in *Populus*	ABA transport demonstrated in *Arabidopsis*, not *Populus*	[65]
AtNPF4.6/NRT1.2/AIT1	Functions as an ABA importer	Plasma membrane	Its ABA-import function was demonstrated in *Arabidopsis*. Homologous NRT1/PTR-family genes occur in *Populus*, but ABA transport by these homologs has not been experimentally established	ABA transport demonstrated in *Arabidopsis*, not *Populus*	[66,67]
PtNRT1/PTR family	Large membrane-transporter family with diverse substrate functions	Membrane proteins; individual localization varies	Sixty-eight NRT1/PTR genes were identified in the *P. trichocarpa* genome with tissue-specific expression patterns; their specific function as ABA transporters was **not demonstrated**	Genomic evidence in *Populus*; ABA-transport function unconfirmed	[67]
Xylem-mediated ABA translocation	Provides a long-distance route for ABA movement and delivery to shoot tissues during water stress	Xylem sap/vascular system	Water stress increases ABA concentrations in poplar xylem sap, and changes in xylem ABA are associated with stomatal responses; ABA flux into leaves has also been quantified in *P. nigra*	Direct physiological evidence in *Populus*	[15,68]

After biosynthesis, ABA needs to be proficiently transported to target tissues to activate the downstream response to drought [69]. ABA can be transported via two different mechanisms, either passive diffusion or active movement by transporter proteins across cellular membranes [70]. But this passive diffusion does not explain the fast and directional transport of ABA in drought-stressed plants, indicating that active transporters are important. Although *Populus* genomes contain numerous genes belonging to the ABC and *NRT1/PTR* transporter families, their specific functions in ABA transport remain poorly characterized. In *Arabidopsis thaliana*, members of these families, including *AtABCG25* and *AtABCG40*, have been functionally demonstrated to mediate ABA export and uptake, respectively. Homologous *NRT1/PTR* genes have been identified in *Populus*, but their roles in ABA transport require further functional validation [63,65,67]. ABA is synthesized in both roots and leaves, and local ABA production in leaf mesophyll cells may be responsible for fast drought responses. ABC transporters are required for the export of ABA from these cells into the apoplast, where it is sensed by the guard cells and triggers stomatal closure. In addition to being produced in the root, ABA produced in the leaves could also travel to other tissues in the plant through the phloem and play a role in whole-plant drought responses [32]. NPF transporters allow the movement of ABA across plasma membranes, thereby regulating its precise distribution among roots, leaves, and other tissues. In addition, variations in transporter expression patterns have been reported across species of *Populus* and in experimental systems, indicating that the regulation of ABA transport may also differ significantly depending on genotype and the severity of drought stress.

In *Populus*, under drought conditions, ABA originating from the roots can be transported to leaf tissues through the xylem, where it contributes to stomatal closure and regulation of water loss [71]. Leaves also have the ability to produce ABA locally under drought conditions, in addition to the ABA that comes from the roots. Root responses to drought can then result from ABA moving towards the roots from the leaves, and this long-distance ABA signaling contributes to the coordination of root development and water acquisition. This bidirectional transport of ABA emphasizes the integrated and dynamic nature of plant drought response that is coordinated by the spatial and temporal control of hormonal signals [9]. Although significant progress has been made in knowledge of ABA biosynthesis and transport, there are still key questions that need to be addressed concerning the integration of ABA signaling networks, long-term adaptation to drought in woody forest species, and ABA responses under changing field conditions. The responses the *Populus* can give to drought can be divided into the following two categories: rapid short-term responses and longer-term adaptive responses. Rapid responses, such as stomatal closure, occur within hours and serve as instant protective mechanisms to minimize water loss. However, this strategy is often accompanied by a temporary decline in photosynthetic activity due to restricted CO_2_ uptake [32,72]. Conversely, long-term adaptive responses change over days to weeks and comprise structural and physiological modifications, including alterations in root architecture, leaf morphology, and growth patterns. These responses contribute to drought acclimation and enhance subsistence under prolonged water deficit conditions.

## 4. ABA-Mediated Molecular Regulation, Hormonal Crosstalk, Multi-Omics Integration, and Epigenetic Control of Drought Responses in *Populus*

**Figure 3:**
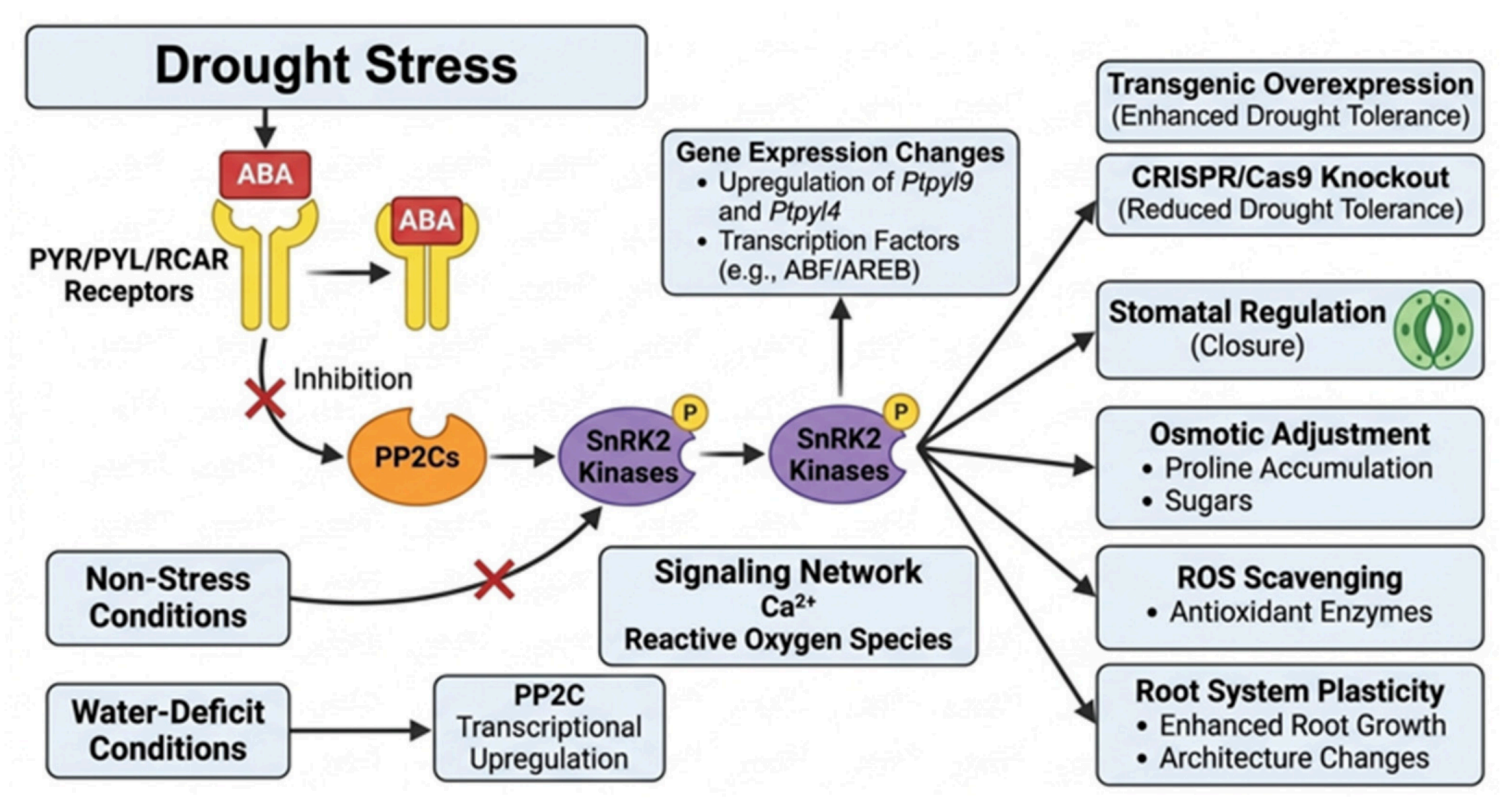
Molecular framework of ABA-mediated drought signaling in *Populus*, highlighting *PYR*/*PYL*/*RCAR*-mediated ABA perception, PP2C inhibition, *SnRK2* activation, downstream transcriptional regulation, and Ca^2+^/ROS-associated signaling.Black arrows indicate signaling or activation, red X symbols indicate inhibition, and P denotes phosphorylation. Created in https://BioRender.com (accessed on 8 June 2026).

In addition to its known roles in the regulation of physiological drought responses, ABA acts as a hub molecular regulator that coordinates environmental perception, transcription regulation, hormonal interactions, metabolic changes and epigenetic reprogramming in *Populus* (Figure 3). The complex network of cellular and molecular processes regulated by ABA signaling has roles in drought acclimation and survival under water-limited conditions [43]. The ABA-mediated drought response includes highly conserved signaling networks that include receptor perception, kinase activation, transcription regulation, and downstream drought stress-responsive processes [73]. Knowledge of these regulatory networks and the interactions among their components offers important insights into the molecular basis of drought tolerance and potential targets for drought resilience using biotechnological approaches (Table 3).

ABA signaling is not autonomous but is involved in many cross-talk events with other hormonal and signaling pathways. Crosstalk between ABA and hormones like Auxin, Ethylene, Cytokinins and Brassinosteroids provides an integrated regulatory framework that balances growth maintenance and drought adaptation [74]. For instance, ABA inhibits the signaling of cytokinin, which helps to reduce growth-promoting processes and reroute resources to stress survival. Likewise, ABA and ethylene interactions may be either antagonistic or synergistic, depending on developmental stage and drought intensity, and impact growth regulation and senescence. Moreover, the ABA signaling is tightly coupled to the secondary messenger pathway, especially Ca^2+^ and ROS. The activation of *SnRK2* kinases leads to NADPH oxidases including RBOH proteins, to produce ROS signals that are involved in downstream stress signaling pathways [75]. ABA-mediated Ca^2+^-signaling also increases drought-responsiveness in cells. Although it is known that there is hormonal and signaling crosstalk, the temporal coordination and hierarchy of these interactions under drought stress are still not well understood in *Populus*.

**Table 3:** Key ABA signaling components associated with drought responses in *Populus* and their relevance for genetic improvement.

Component Type	Representative Gene(s)	Function	Experimental Evidence Under Drought/ABA	Drought-Related Response	Genetic-Engineering Relevance	References
ABA Receptor	*PePYL4*	ABA perception and regulation of downstream ABA signaling.	Functional analysis in transgenic poplar showed that *PePYL4* manipulation altered ABA-associated stomatal responses, water-use efficiency (WUE), and ROS homeostasis under drought	Enhanced drought tolerance was associated with improved WUE and ROS-scavenging capacity.	Experimentally supported target for improving drought tolerance through ABA-mediated water conservation and antioxidant responses.	[76]
Negative Regulator	*PePP2C* family members	PP2Cs negatively regulate ABA signaling, principally through inhibition of SnRK2 activity	Genome-wide characterization of *P. euphratica* PP2Cs revealed differential expression of individual family members under multiple abiotic stresses	Distinct stress-responsive expression patterns indicate functional differentiation among *PePP2Cs*.	Provides candidate negative regulators for functional manipulation; direct engineering effects require further validation.	[77]
Protein Kinase	*PtSnRK2* family members	Phosphorylate downstream ABA-responsive proteins and transcription factors after release from PP2C inhibition.	Poplar *SnRK2* genes showed differential transcriptional responses to ABA and abiotic stresses	Demonstrates gene-specific *SnRK2* responses to environmental and hormonal signals.	Provides specific *SnRK2* candidates for subsequent functional engineering.	[78]
Protein kinase	*PeSnRK2* family	Positive regulators of ABA signaling downstream of the *PYL*-PP2C module.	Genome-wide identification and expression profiling demonstrated differential responses of *PeSnRK2* genes to abiotic stresses.	Stress-responsive expression supports the involvement of PeSnRK2s in ABA-associated stress signaling.	Candidate genes for functional validation and manipulation of ABA-dependent drought responses	[79]
Transcription Factor	*PtrAREB1-2*	ABRE-binding ABA-responsive TF regulating downstream drought-response genes.	Molecular assays demonstrated regulation of *PtrNAC006*, *PtrNAC007* and *PtrNAC120* by *PtrAREB1-2*. Altering *PtrAREB1-2* expression strongly affected drought survival.	Overexpression substantially enhanced drought tolerance, whereas RNAi suppression increased drought sensitivity	Direct functional evidence: overexpression improves drought tolerance, although growth effects indicate a possible growth-stress tolerance trade-off.	[80,81]
Ion-channel signaling	SLAC1/GORK-associated pathway	Regulates guard-cell anion and K+ efflux during ABA-induced stomatal closure.	ABA-ROS-Ca^2+^ signaling regulates guard-cell ion channels during drought-induced stomatal closure.	Promotes stomatal closure and thereby limits transpirational water loss.	Provides a mechanistic basis for manipulating stomatal water-use traits; direct *Populus* engineering evidence remains limited.	[82]
Secondary messenger signaling	CBL-CIPK pathway	Ca^2+^ sensor-kinase modules integrate Ca^2+^ signals with ABA and abiotic-stress signaling	CBL-CIPK networks participate in abiotic-stress responses, ion homeostasis, and hormone crosstalk.	Contributes to stress-responsive signaling and ion-transport regulation.	Potential downstream targets for manipulating drought signaling; direct *Populus*-specific engineering evidence requires validation.	[83]

### 4.1. ABA Signaling Network and Molecular Components

The basic ABA signal transduction system in *Populus* is composed of three well-conserved components: *PYR*/*PYL*/*RCAR* receptors, PP2C phosphatases, and *SnRK2* kinases that act in concert to control the expression of stress-responsive genes downstream in the pathway [84,85]. This signaling cascade is activated by drought- induced accumulation of ABA, which is primarily controlled by the activity of enzymes named 9-cis-epoxycarotenoid dioxygenase (*NCED*). In *Populus*, expression profiling of ABA biosynthesis and signaling component genes showed that they are dynamically regulated during dehydration, indicating that modulation of the ABA pathway is crucial for drought adaptation [75,86]. Soluble cytoplasmic receptors that belong to the *PYR*/*PYL*/*RCAR* family mediate ABA perception as primary molecular sensors of ABA signaling [87]. The binding of ABA causes conformational changes in the *PYR*/*PYL*/*RCAR* receptor that allow for interaction with PP2C phosphatase which triggers activation of downstream signaling. Genomic analysis showed the presence of 14 genes coding for the *PYR*/*PYL* receptor family in *P. trichocarpa*, including two genes, *PtPYL9* and *PtPYL4*, that were highly drought-responsive and were induced to play a role in drought-responsive gene regulation and ABA sensitivity [88]. In other plant systems, genetic manipulation of *PYR*/*PYL* receptor function has also been shown to increase drought tolerance which highlights the key role of ABA perception for adaptation to drought stress [89]. In addition, heterologous expression of poplar ABA receptor genes (*PtPYRL1* and *PtPYRL5*) in *Arabidopsis thaliana* enhanced ABA sensitivity and drought tolerance, demonstrating the conserved functional capacity of poplar ABA receptors in ABA signaling [88,90]. Further studies in transgenic poplar plants showed that overexpression of *PtPYRL1* and *PtPYRL5* improved drought tolerance through enhanced ABA responses, including increased stomatal closure and reduced water loss [91,92]. The use of genome editing techniques, such as CRISPR/Cas9, is, however, still difficult in woody plants like *Populus* because of their long generation time, polygeny, and stable transformation and field evaluation.

PP2C phosphatases are the key negative regulators of ABA signaling and act to inhibit the activity of the downstream *SnRK2* kinases in non-stress conditions [93]. Under drought stress, ABA-bound *PYR*/*PYL*/*RCAR* receptors bind to and inhibit the activity of PP2Cs, allowing activation of *SnRK2* kinases. The *Populus* ABI1- and ABI2-like genes have been characterized as PP2C homologs and have been found to be transcriptionally responsive to water-deficit conditions [94]. In addition, silencing of PP2C genes in *P. euphratica* by RNA interference increased ABA sensitivity and drought survival, underscoring the important regulatory function of PP2Cs in regulating ABA-dependent drought responses [95,96]. However, ABA sensitivity and drought tolerance vary between *Populus* genotypes, suggesting that genetic background, experimental conditions, and environmental factors play important roles in the contribution of ABA signaling components.

When released from PP2C-mediated inhibition, *SnRK2* (Sucrose Non-Fermenting-1-Related Protein Kinase 2) kinases serve as central signaling hubs that relay ABA signals through the phosphorylation of a variety of substrates such as transcription factors, ion transport regulators, and stress-associated proteins [97]. There are 12 *SnRK2* genes in *P. trichocarpa*, and most are drought-responsive: subclass II *SnRK2* genes are broadly expressed in leaves, stems, and roots, while subclass III genes are more highly expressed in leaves [78]. Transcriptomic analyses in *Populus* have revealed extensive reprogramming of drought-responsive transcriptional networks, whereas phosphoproteomic studies in *A. thaliana* plants have demonstrated that ABA-activated *SnRK2* kinases phosphorylate downstream targets involved in stress signaling, providing mechanistic insights into conserved ABA signaling processes [32,98]. Together, these findings suggest that conserved ABA signaling mechanisms contribute to drought adaptation in *Populus*, although comprehensive phosphoproteomic characterization of ABA-mediated drought responses in *Populus* remains limited. The phosphorylation events activate the ABA-responsive transcription factors such as AREB/ABF, NAC, MYB, and AP2/ERF family proteins that control the general drought-responsive gene expression programs. In *Populus*, integrated transcriptomic analysis of the plants under drought has identified important genes related to ABA-associated hormone signaling and molecular adaptation [99,100]. However, the majority of transcriptomic/phosphoproteomic studies have been conducted under short time frame-controlled drought stress, and it is unknown what occurs in ABA signaling under natural drought field conditions for long periods of time.

Along with the known *PYR*/*PYL*–*PP2C*–*SnRK2* pathway, there are other cellular regulatory mechanisms that affect the complexity of ABA signaling in woody plants. For example, the ubiquitin E3 ligase, *PeCHYR1*, of *P. euphratica*, has a drought tolerance role that is related to ABA [42]. In combination, these discoveries reveal that ABA regulation in woody species is not limited to the classic signaling module and highlight the need for a combination of genetic, multi-omics, and field-based methods to further decipher ABA regulatory networks in *Populus*. Table 4 provides an overview of major ABA-related molecular and physiological responses found in *Populus* under drought conditions.

### 4.2. ABA-Driven Hormonal Crosstalk During Drought Adaptation

**Figure 4:**
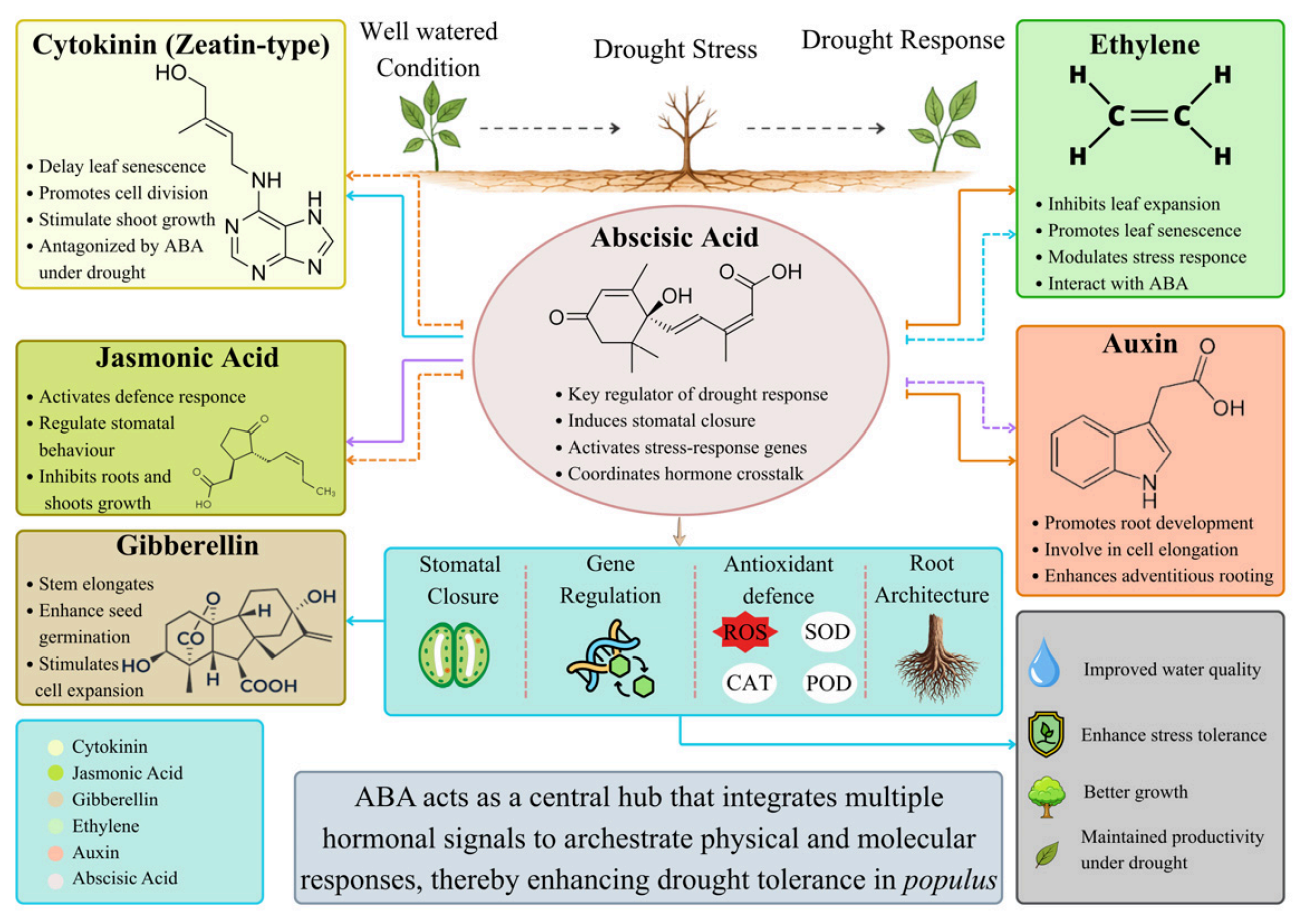
ABA-centered hormonal crosstalk during drought adaptation in *Populus*, highlighting interactions with auxin, ethylene, cytokinins, jasmonic acid, and gibberellins and their roles in root development, stomatal regulation, antioxidant defense, and growth responses. Solid lines with arrows indicate direct positive regulation or activation, blunt-ended lines represent inhibition or negative regulation, and dashed lines denote stress-induced or indirect regulatory cross-talk. Created in https://BioRender.com (accessed on 8 June 2026).

ABA signaling is not the only hormone involved in plant response to drought. While ABA is a key regulator in drought responses, its physiological impacts are influenced by cooperation and/or competition with auxin, cytokinins, ethylene, jasmonic acid (JA), gibberellins (GAs) and other signaling molecules [102,103]. Such interactions allow plants to balance stress protection with growth and development and the results of these interactions depend on the tissue and developmental phase, as well as the severity and duration of the drought [104]. In *Populus*, ABA-mediated crosstalk is involved in a coherent regulation of root-system architecture, stomatal behavior, growth, senescence, the regulation of antioxidant defense and the water-use efficiency, thus balancing water conservation and carbon acquisition in the production of biomass [12]. ABA signal transduction is also coupled to the secondary messengers, especially Ca^2+^ and reactive oxygen species (ROS), that link hormonal perception to ion transport, redox homeostasis, stomatal movements and later signaling networks [83]. Therefore, it is important to think of ABA as a hub in a complex signaling network during drought, and not as a stand-alone drought-response pathway. The most important ABA’s interactions with other phytohormones in *Populus* drought adaptation are summarized in Figure 4 and discussed below.

#### 4.2.1. ABA–Auxin Crosstalk

The root system architecture is one of the important plant developmental traits involved in adaptations to dry conditions, and auxin plays a critical role in regulating the plant growth and developmental responses [102]. ABA triggers massive changes in the distribution and signaling of auxin in *Populus* roots and implements a suitable root development strategy under drought to help the plants access water from deeper and dryer soil layers via lateral root formation, and to reduce primary root elongation. ABA signaling targets auxin transporters (e.g., PIN) thus controlling auxin flow and altering root growth during drought [105]. In *Populus*, the *PdNF*-*YB21*–*PdFUS3*–*PdNCED3* pathway connects ABA and auxin signaling by facilitating auxin transport to root tips, thereby promoting root growth under drought conditions. In addition, ABA-mediated regulation of root development is context-dependent and varies with ABA signaling status and environmental conditions, allowing plants to balance root elongation and lateral root formation during water limitation [106]. In a similar way, the development of lateral roots in *Populus* is controlled by ABA–auxin crosstalk. *PeFUS3* under drought conditions connects ABA signaling to auxin transport by regulating the ABA receptor *PePYL3* and auxin transport genes *PIN2*, *PIN6a*, and *AUX1*, and thus enhances drought tolerance by promoting the growth of lateral roots [9]. Thus, ABA-auxin crosstalk in *Populus* should be considered as a dynamic process to coordinate root elongation and branching under the influence of ABA and auxin levels, and drought intensity, rather than as a switch from primary to lateral root growth. This coordinated hormonal control increases root-system plasticity and could help to access spatially heterogeneous soil-water resources during drought [106].

#### 4.2.2. ABA–Ethylene Crosstalk

Ethylene–ABA interactions under drought stress can be either antagonistic or synergistic depending on tissue type, developmental stage, and stress duration. Ethylene is often associated with senescence progression, whereas ABA promotes stomatal closure and water conservation during early drought responses [107]. During prolonged drought, ABA and ethylene signaling may interact to regulate senescence-related processes and resource allocation, contributing to stress adaptation [108]. In *P. nigra*, drought-induced changes in ABA and ethylene levels under heterogeneous water availability suggest that hormonal interactions contribute to the regulation of stress-responsive processes [109]. Transcriptomic and functional analyses have further highlighted the role of ethylene-responsive transcription factors in regulating drought tolerance in poplar [110]. Overall, ABA-ethylene crosstalk represents a dynamic regulatory mechanism that coordinates growth maintenance, senescence progression, and stress survival according to drought intensity, duration, and developmental context [108].

#### 4.2.3. ABA–Cytokinin Crosstalk

ABA-cytokinin interactions generally exhibit antagonistic regulation during drought stress by balancing growth maintenance and stress survival. In *P. trichocarpa*, ABA-associated regulation of root development contributes to drought adaptation through modulation of stress-responsive pathways, including lateral root formation [111]. Under well-watered conditions, cytokinins promote cell division, shoot growth, and delayed senescence [73]. In other plant species, drought-induced ABA accumulation has been shown to suppress cytokinin biosynthesis and signaling, thereby restricting growth-promoting processes and redirecting resources toward stress defense [112]. Recent multi-omics study further demonstrates that ABA-cytokinin crosstalk coordinates the trade-off between stress defense and growth maintenance during drought adaptation, where ABA primarily enhances stress-responsive pathways while cytokinins contribute to growth regulation [113,114,115]. Reduced cytokinin activity may contribute to growth inhibition and altered developmental responses during drought stress. However, the magnitude and direction of ABA-cytokinin interactions vary among genotypes and depend on drought severity, developmental stage, and tissue-specific hormonal regulation [113].

#### 4.2.4. ABA–Jasmonic Acid (JA) and ABA–Gibberellin (GA) Crosstalk

ABA also interacts with other phytohormones such as jasmonic acid (JA) and gibberellins (GA) to coordinate drought adaptation, including stress protection and growth regulation [116]. JA–ABA crosstalk was found to improve drought tolerance by the PeJAZ2-PeOST1 regulatory module that facilitates stomatal closure in response to ABA and the signaling response to H_2_O_2_ in *P. euphratica* [22]. Recent studies in *Populus* have shown that the JA signaling components can interact with ABA-associated pathways to control drought tolerance, such as modifying stomatal responses and stress-responsive transcriptional networks [117]. By contrast, ABA–GA interactions are typically related to growth–stress compromises under drought conditions. An increase in ABA levels is often associated with a decrease in GA mediated growth promoting processes that either reduce GA biosynthesis or the activity of GA signaling, so that resources are diverted from growth to stress survival. An appropriate balance between ABA and GA signaling is important, however, because too much growth inhibition can have a negative impact on recovery and on long-term productivity in response to repeated droughts [118,119].

In *Populus*, ABA–JA and ABA–GA interactions work together to broaden the function of ABA beyond water conservation to include coordination of defense activation, growth regulation and drought recovery. These hormonal interactions are very situation-dependent, depending on the stress level, the time of the stress, the type of tissue, the stage of development and the genotype. The way that they coordinate with each other during a long period of drought, however, and under field conditions in mature *Populus* trees, is not yet well understood.

### 4.3. Multi-Omics Dissection of ABA-Regulated Drought Responses

ABA signaling has been shown to involve the mediation of biomass trade-offs and biomass allocation under drought stress in *Populus* and other plants, and recent omics approaches have enhanced the understanding of drought tolerance mechanisms in these species [120]. In the case of *Populus*, the integrative approaches yield insights into the molecular mechanisms underlying the ABA-dependent control of drought resistance and reveal important regulatory genes, proteins, and metabolites, as well as epigenetic modifications (Table 5). The combination of omics technologies and epigenetic analyses can provide valuable insights into ABA-mediated drought responses and potential new targets for genetic improvement and sustainable forestry management in *Populus.* Despite the large volume of omics data currently available, integrating transcriptomic, proteomic, metabolomic, and epigenomics datasets into unified regulatory models remains a major challenge in *Populus* drought research.

#### 4.3.1. Transcriptomics

Transcriptomics has now become an indispensable technique for dissecting the molecular mechanisms underlying drought tolerance response mediated by ABA, and global gene expression profiling has been used to profile such gene expression dynamics [121]. In *Populus*, drought-induced genes involved in ABA signaling and related pathways have been identified by RNA-seq analyses, such as osmotic adjustment, ROS homeostasis, aquaporin-mediated water transport and hormonal regulation [122]. Further, transcriptomic profiling shows differential regulation of various families of transcription factors, such as NAC, MYB and ABF/AREB, that act as regulators of ABA-responsive and other drought associated genes [123,124]. However, transcriptomic study of genetically modified *Populus* has aided in correlating changes in ABA-related regulatory genes with more general drought-responsive transcriptional networks, such as in *PdNCED3*-overexpressing and RNAi lines, which exhibited changes in ABA accumulation and ABA-responsive gene expression [7]. But transcript abundance does not always equal the abundance of proteins or metabolites. Bogeat-Triboulot et al. (2007) [125] observed in *P. euphratica* that protein profile responses to drought were more pronounced than changes in transcription, and that there was no consistent relationship between transcript abundance and protein abundance.

Similarly, integrated transcriptomic and metabolomic analyses of drought-stressed poplar revealed genotype dependent differences and the extent of transcriptomic responses was not necessarily dependent on the extent of metabolite abundance changes [126]. Thus, combining transcriptomic data with proteomic, metabolomic and physiological measurements will enable a more comprehensive basis for the identification of functionally relevant drought-response mechanisms and potential targets for the improvement of drought resistance in *Populus*.

#### 4.3.2. Proteomic and Post-Translational Modifications (PTMs)

Proteomic approaches provide complementary information to transcriptomic analyses by revealing drought-induced changes at the protein level, including alterations in protein abundance, phosphorylation patterns, and other post-translational modifications (PTMs) that regulate stress-responsive pathways. Unlike transcriptomic analyses, which reflect changes in gene expression, proteomic and PTM-based approaches provide insights into protein-level regulation related with ABA signaling, metabolism, and stress adaptation [127,128]. In *Populus*, functional studies have demonstrated that ABA signaling components, including PP2C proteins and ABA receptors, contribute to drought responses. For example, the PP2C homolog *PeHAB1* from *P. euphratica* interacts with the ABA receptor PYL4 and acts as a negative regulator of ABA signaling, highlighting the importance of PP2C–PYL regulatory modules in drought adaptation [129]. Phosphorylation is one of the most extensively studied PTMs involved in ABA-mediated drought signaling. Phosphoproteomic analyses have revealed dynamic ABA-induced phosphorylation landscapes, identifying numerous proteins involved in signal transduction, transcriptional regulation, and stress responses [93]. In particular, *SnRK2* kinases function as central phosphorylation hubs in ABA signaling by modifying downstream targets, including ABF/AREB transcription factors and other stress-associated proteins, thereby activating ABA-responsive drought responses [98]. These findings demonstrate that phosphorylation-mediated regulation provides a rapid mechanism for transmitting ABA signals beyond transcriptional changes. Although these phosphoproteomic studies were primarily conducted in *Arabidopsis*, they provide important mechanistic insights into conserved ABA signaling processes that may contribute to drought adaptation in woody species such as *Populus*.

Beyond phosphorylation, ubiquitination represents another important PTM involved in ABA signaling regulation. Ubiquitination controls protein stability and activity by targeting specific regulatory proteins for degradation, thereby modulating the intensity and duration of stress signaling pathways [101]. In *Populus*, recent evidence demonstrated that the ubiquitin E3 ligase *RZFP1* enhances drought tolerance by promoting the degradation of PP2C-9, a negative regulator of ABA signaling, thereby influencing ABA pathway activity and drought adaptation [95]. These findings highlight that ubiquitin-mediated regulation provides an additional layer of control over ABA signaling components in *Populus*. Despite significant progress, the spatiotemporal regulation and functional contribution of many drought-associated PTMs under variable field conditions remain insufficiently understood, particularly in *Populus*. Integrating proteomic and PTM analyses with transcriptomic, metabolomic, and physiological datasets will be essential to identify regulatory mechanisms that contribute to ABA-mediated drought adaptation in *Populus*. In addition to protein-level regulation, metabolomic analyses provide further insights into the biochemical adjustments associated with ABA-mediated drought responses.

#### 4.3.3. Metabolomics

In drought-stressed poplars, significant metabolic changes have been recorded for metabolomics reflecting metabolic reprogramming, such as osmolyte accumulation and antioxidants, which can help in drought acclimation. Metabolomic studies have shown that drought stress in poplar species is associated with the accumulation of osmolytes such as proline and glycine betaine, together with antioxidant-related metabolites, including ascorbate and glutathione, reflecting adaptive metabolic responses to water deficit [130]. These metabolic changes help in maintaining cellular osmotic balance, redox balance, and protection from drought-induced oxidative damage. Understanding the coordinated regulation of genes in response to heat and drought is important in terms of transcriptomic analyses conducted in poplars, which have shown overlapping heat and drought-responsive genes [121]. Combination of transcriptomic and metabolomic studies can be used to gain insight into drought adaptation regulatory network and metabolic pathways which can be targeted for future breeding and biotechnology applications with the help of ABA. In addition, metabolic changes are not consistent across different *Populus* species or environmental conditions, making it difficult to identify universal drought-resilience biomarkers. Multi-omics data collected under field-relevant drought conditions will be required for validation of candidate biomarkers to help to better predict drought resilience.

### 4.4. Epigenetic Regulation and Stress Memory

Changes in chromatin accessibility and stress-responsive gene expression, via epigenetic modifications like DNA methylation, histone modifications, and regulatory small RNAs, add a further layer of control over ABA-associated drought responses, which do not involve any changes in DNA sequence [131]. Drought stress causes changes in DNA methylation in *P. trichocarpa*, including methylation changes in different genomic regions and for various types of methylation, and methylation is correlated with alterations in expression of drought-responsive genes. But the exact relationship of the methylation changes induced by drought to stress-responsive transcription is not fully understood [132]. These results suggest that methylation of DNA is one of the key aspects of the regulation of transcription in *Populus* in association with drought. Similar drought-associated DNA methylation responses have also been reported in other plant systems. In tomato, drought stress was shown to induce DNA methylation changes in the promoter region of the water stress-responsive *Asr2* gene, demonstrating that epigenetic regulation of stress-responsive loci represents a conserved mechanism contributing to drought adaptation across plant species [133]. Furthermore, the drought-induced change in acetylation has been shown to be connected with ABA-dependent regulation of gene expression, via the regulatory coupling between AREB1, ADA2b, and GCN5 in *P. trichocarpa* by Li et al. (2019) [81]. In addition, *PtrAREB1-2* binds to ABA-responsive elements (ABREs) in the promoters of *PtrNAC006*, *PtrNAC007* and *PtrNAC120* and recruits the ADA2b–GCN5 histone acetyltransferase complex, leading to higher acetylation of H3K9, enrichment of RNA polymerase II and activation of these drought-responsive genes [81].

Furthermore, genetic manipulation supported the functional importance of this mechanism: overexpression of the components of this pathway resulted in an increase in drought tolerance, while downregulation resulted in an increase in drought sensitivity. ABA signaling dynamics are also controlled by histone modifications. Under drought, the expression of ABA-responsive transcription factors (ABF, NAC) was greatly affected and the promoter of these genes showed drought mediated regulation of histone acetylation, and H3K27ac and H3K4me3 marks were identified by ChIP (chromatin immunoprecipitation) assays [132,134]. Another regulatory mechanism that may regulate stress-responsive transcripts is small RNAs, particularly microRNAs (miRNAs), which may interact with epigenetic regulatory pathways [135]. Drought-induced changes in miRNA expression profiles can influence ABA signaling intensity and drought resilience by regulating stress-responsive gene networks [136]. In addition to small RNAs, long non-coding RNAs (lncRNAs) have emerged as important regulatory molecules involved in plant stress responses by modulating gene expression through transcriptional and epigenetic mechanisms [137]. Although the contribution of lncRNAs and other epigenetic mechanisms to drought memory in *Populus* remains largely unexplored, studies in model plants suggest that previous stress exposure can induce stable chromatin modifications that influence subsequent stress responses. In *Arabidopsis*, repeated dehydration resulted in the formation of transcriptionally “trainable” genes, characterized by increased levels of Ser5-phosphorylated RNA polymerase II and H3K4me3 during recovery, which promoted enhanced transcription during subsequent dehydration events [138]. Later studies showed that H3K4me3 and H3K27me3 modifications were associated with dehydration-memory genes in a gene-specific manner [139]. Future research in forest trees should determine whether drought-induced changes in DNA methylation, histone modifications, lncRNAs, or small-RNA profiles are maintained during recovery, repeated drought exposure, seasonal growth cycles, or subsequent generations.

**Table 5:** Omics and epigenetic insights into ABA-mediated drought responses in *Populus* and related plant systems.

Omics Type	Key Findings	Methodologies	Evidence Source	References
Transcriptomics	Genome-wide transcriptomic analysis identified ABA- and drought-responsive R2R3-MYB transcription factors in *P. euphratica*	RNA-Seq, qRT-PCR	*Populus*-specific study	[13]
Transcriptomics	Genome-wide transcriptome analyses identified ABA-responsive drought-regulated genes, including transcription factors involved in stress adaptation and ABA signaling pathways.	RNA-seq/microarray	Conserved ABA drought-response mechanisms in *Arabidopsis*	[140]
Phosphoproteomics	ABA-dependent phosphorylation of *SnRK2* targets, including ABF/AREB transcription factors and stress-associated proteins, revealed in *Arabidopsis*.	LC-MS/MS phosphoproteomics	Conserved ABA signaling evidence from *Arabidopsis*	[93,98]
Proteomics/PTM analysis	Ubiquitin-mediated degradation of PP2C-9 regulates ABA signaling and drought tolerance in *Populus*	Protein interaction, ubiquitination analysis	*Populus*-specific study	[95]
Metabolomics	ABA-associated accumulation of osmoprotectants, including proline, betaine, and soluble sugars during drought stress	GC-MS, LC-MS, HPLC	*Populus*-based drought studies	[130,141]
Epigenomics	Differential DNA methylation associated with ABA biosynthesis and stress-responsive genes	Bisulfite sequencing, ChIP-qPCR	*Populus*-specific evidences	[133,142]
Epigenomics	Drought stress induces DNA methylation changes in stress-responsive genes, revealing an epigenetic layer associated with drought adaptation	Bisulfite sequencing, methylation analysis	Epigenetic regulation of drought responses demonstrated in tomato	[143]
Small RNA Analysis	miRNAs regulate ABA-related targets, including *ABFs*, *SnRK2s*, and *PP2Cs*	miRNA-seq, degradome sequencing	*Populus*-based drought study	[144]

## 5. Biotechnological Interventions and Forest Resilience

Biotechnological methods are valuable tools to characterize and manipulate drought-responsive pathways in *Populus*. The use of conventional genetic approaches, such as gene overexpression and RNAi, has allowed direct assessment of genes involved in drought- and ABA-associated signaling, and new genome editing technologies have provided more precise ways to manipulate stress-responsive regulatory networks [9,145].

### 5.1. Genetic Modification Using Gene Overexpression and RNAi

Genetic modification approaches, particularly gene overexpression and RNA interference (RNAi)-mediated silencing, have become fundamental strategies for elucidating the functional roles of stress-responsive genes in plants. Overexpression approaches provide a gain-of-function framework by enhancing the expression of candidate genes, allowing researchers to evaluate their potential contributions to abiotic stress tolerance and associated physiological responses [146,147]. Conversely, RNAi-mediated suppression serves as a loss-of-function approach by reducing transcript abundance, enabling functional validation of genes involved in stress adaptation and signaling pathways [148]. The complementary application of these strategies has substantially improved the identification and characterization of regulatory genes involved in drought adaptation, hormone signaling, reactive oxygen species (ROS) regulation, and stress-responsive metabolic pathways across plant species [147].

To elucidate the functions of drought-responsive genes in *Populus*, both gene overexpression and RNAi-mediated silencing have proven to be widely used techniques. A particularly relevant example is *DREB46* (*P. trichocarpa*), which is highly responsive to both ABA and osmotic stress conditions related to drought [149]. The overexpression of *DREB46* significantly improved drought survival and was associated with increased SOD, POD, and CAT activities, higher proline levels, and reduced ROS accumulation and lipid peroxidation, and an increase in adventitious-root development [149,150]. The RNAi-mediated suppression of *DREB46*, on the other hand, resulted in the opposite drought-sensitive phenotype. The *DREB46* transcription factor was also demonstrated to interact with the PP2C47 phosphatase, which may be a molecular link between this drought-responsive transcription factor and ABA-associated regulatory signaling [149,151]. These results demonstrate the power of complementary gain and loss of function studies to identify the role that individual regulatory genes play in drought adaptation of *Populus*. However, transgenic trees or RNAi-mediated modifications can result in context-dependent effects on growth and development to constitutive changes in stress-responsive pathways, and evidence remains limited regarding the long-term stability and ecological performance of transgenic or RNAi-modified trees under field conditions [150]. Gene overexpression and RNAi are thus still useful for functional validation purposes and more precise genome-editing approaches are discussed in the subsequent sections.

### 5.2. CRISPR/Cas9 Genome Editing: Precision Targeting of ABA Pathways

CRISPR/Cas9 genome editing has become a powerful tool for dissecting and manipulating genes involved in plant abiotic stress responses. Studies in crop species have demonstrated that targeted modification of drought- and stress-responsive genes can alter regulatory pathways associated with ABA signaling, transcriptional regulation, stomatal function, and cellular stress responses [152,153]. For example, CRISPR/Cas9-mediated editing of *OsDST* improved drought and salt tolerance-related traits in rice, while modification of *ARGOS8* in maize enhanced drought tolerance by improving yield performance under drought stress conditions [154,155]. These examples establish the potential of genome editing for modulating stress-responsive regulatory networks; however, their direct translation to woody plants remains limited. Recently, CRISPR/Cas9 approaches have been successfully applied in *Populus* to improve abiotic stress tolerance. Zhang et al. (2023) identified *PagHyPRP1* (*PagHyPRP1A* and *PagHyPRP1B*) as negative regulators of drought and salt stress responses in hybrid poplar (*Populus alba* × *P. glandulosa*) and used CRISPR/Cas9-mediated gene editing to generate targeted mutants with enhanced stress tolerance, providing a promising strategy for improving drought and salt resilience in woody plants [145]. Additionally, CRISPR/Cas9-mediated editing of two base pairs in the core promoter region of *PagHCF106* in hybrid poplar (*P. alba* × *P. glandulosa*) reduced stomatal opening, reduced water loss, and altered H_2_O_2_ accumulation in guard cells, highlighting the potential of promoter engineering for enhancing drought tolerance in poplar [156].

**Figure 5:**
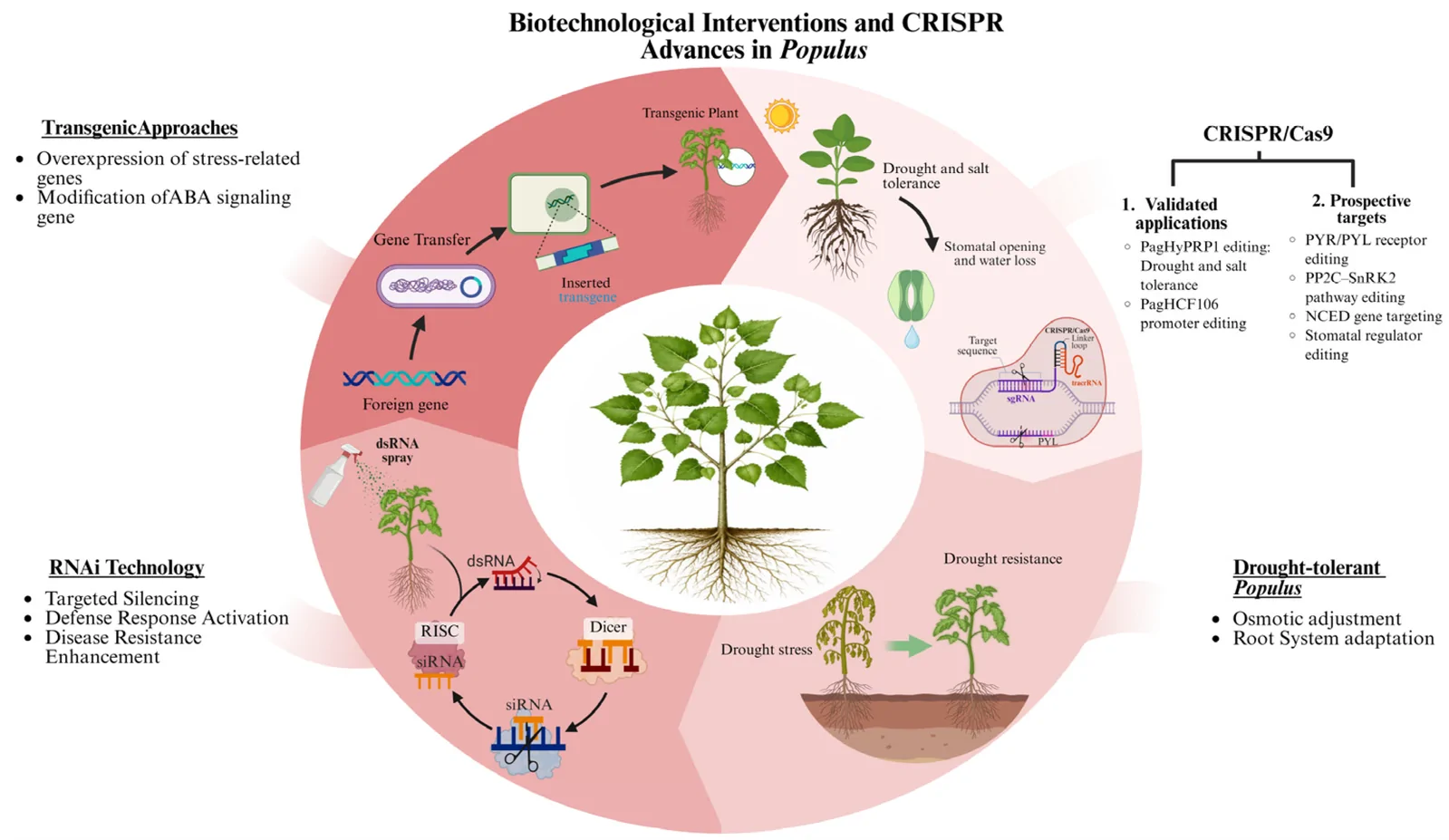
Biotechnological strategies for enhancing drought resilience in *Populus*, integrating transgenic approaches, RNAi-mediated gene silencing, and CRISPR/Cas genome editing to modulate ABA signaling, stomatal regulation, osmotic adjustment, and root-system adaptation. Solid arrows indicate the sequential direction of molecular and cellular processes (such as gene transfer, RNA interference pathways, and CRISPR editing steps), while distinctly colored background segments delineate the different methodological categories. Created in https://BioRender.com (accessed on 8 June 2026).

Overall, these studies provide direct evidence that CRISPR/Cas9 can be used not only for functional characterization but also for targeted improvement of stress resilience traits in *Populus* (Figure 5). However, the application of CRISPR/Cas9 in woody perennials remains more challenging than in annual crops due to their large and complex genomes, long generation cycles, genotype-dependent transformation efficiency, regeneration limitations, and difficulties associated with long-term field [157,158]. Although ABA signaling components, particularly the *PYR*/*PYL*–*PP2C*–*SnRK2* regulatory module, have been extensively characterized in model plants, their direct manipulation through genome editing for improving drought tolerance in forest trees remains relatively unexplored [159]. Future studies should therefore focus on identifying and validating ABA-related genome-editing targets across diverse *Populus* genotypes to develop reliable strategies for enhancing drought resilience and climate adaptation in woody species.

### 5.3. Advanced CRISPR-Based Strategies for Engineering Drought Resilience in Populus

In addition to traditional single-gene editing, new opportunities for precise manipulation of complex regulatory networks involved in drought adaptation exist with advanced CRISPR-based methods, such as multiplex genome engineering, CRISPR activation (CRISPRa), and epigenome editing. Multiplex CRISPR/Cas systems can be used to introduce multiple modifications or regulate multiple genes simultaneously, which is particularly useful when targeting interconnected pathways like ABA biosynthesis, perception and signaling [160]. ABA-related genes, including those involved in ABA biosynthesis (*NCED*), ABA receptors (*PYL*), and downstream signaling components (PP2C–*SnRK2* modules), are coordinately regulated and represent promising targets to enhance drought-responsive traits in plants [84,161]. However, multiplex editing is technically challenging since the functional consequences of changing multiple regulatory genes at once could be hard to predict and extensive validation is necessary to ensure there are no unexpected effects on growth, development, or stress adaptation [162]. This limitation is particularly relevant for woody plants such as *Populus*, where transformation efficiency, regeneration capacity, and long generation cycles restrict the application of multi-gene editing approaches [163].

An alternative strategy is to increase the expression of endogenous genes using CRISPRa, which enhances gene expression without altering the underlying genome sequence. CRISPRa employs a catalytically inactive Cas9 (dCas9) fused with transcriptional activation domains to selectively enhance target gene expression and provides a promising strategy for modulating stress-responsive pathways [164,165]. Likewise, epigenome editing approaches based on CRISPR systems can specifically modify regulatory elements, DNA methylation patterns, or chromatin states to regulate gene expression without changing DNA sequences [166]. These strategies could be particularly valuable for tree species such as *Populus*, given their long generation times and the difficulty of achieving stable genome modifications for developing stress-resilient trees [167]. Further studies are needed for validation of drought-responsive targets, transformation and regeneration systems, stability, ecological performance, and long-term effects of transformed *Populus* genotypes in the field.

## 6. Conclusions and Future Perspectives

The ability to integrate ABA signaling into biotechnology offers potential solutions to enhance drought resistance and forest sustainability in *Populus* under increasingly severe water-limiting conditions. Molecular biology, multi-omics technologies, and genome-editing approaches have significantly deepened our understanding of the mechanisms underlying drought adaptation mediated by ABA and identified key regulatory elements, such as the *PYR*/*PYL*–*PP2C*–*SnRK2* signaling module, downstream transcription factors, hormonal networks, and stress-responsive metabolic pathways. Comprehensive transcriptomic, proteomic, metabolomic, and epigenetic studies have also revealed complex regulatory networks associated with drought responses, although several mechanisms require further functional validation in *Populus*. However, translating these discoveries into improved *Populus* genotypes remains challenging. While many ABA-related mechanisms and biotechnological strategies have been established under controlled experimental conditions, their stability, ecological performance, and long-term effects under field conditions remain poorly understood. In particular, more studies are needed to understand ABA-mediated hormonal crosstalk, the stress-memory mechanisms, and genotype-dependent responses to repeated and fluctuating drought. While CRISPR/Cas9 and other newly developed CRISPR-based regulatory technologies are promising approaches for fine-tuning drought-responsive pathways, their application in woody species remains limited due to low transformation efficiency, long generation time, and the necessity of long-term ecological evaluation.

Future research priorities include integrating molecular engineering, physiological characterization, multi-omics approaches, and field-scale evaluation to make discoveries of ABA relevant to the development of drought-resilient *Populus* cultivars. These strategies will be vital for sustainable forestry, ecosystem stability, and adaptation to future climate change scenarios, and they will involve interdisciplinary approaches.

## Figures and Tables

**Table 4:** Key molecular components and regulatory processes involved in ABA-mediated drought responses in *Populus*.

Regulatory Level	Molecular Mechanism	Representative Components in *Populus*	Role in ABA-Mediated Drought Response	References
ABA biosynthesis and signal initiation	Drought-induced ABA accumulation through regulation of ABA biosynthetic enzymes	NCED family	Initiates ABA-dependent signaling during water deficit and contributes to transcriptional reprogramming associated with drought acclimation	[75,86]
ABA perception	ABA binding induces conformational changes in *PYR*/*PYL*/*RCAR* receptors, enabling interaction with PP2Cs	*PYR*/*PYL*/*RCAR*; *PtPYL4*, *PtPYL9*	Functions as the primary ABA-sensing step and determines sensitivity of downstream ABA signaling	[87,88]
PP2C-mediated negative regulation	PP2Cs inhibit *SnRK2* activity under non-stress conditions; ABA-bound receptors suppress PP2C activity during drought	ABI1/ABI2-like clade A PP2Cs	Releases *SnRK2* kinases from negative regulation and enables activation of ABA signaling	[78,85,97]
*SnRK2* kinase activation	Activated *SnRK2*s phosphorylate downstream regulatory proteins following PP2C inhibition	*SnRK2* family; subclass II and III members	Acts as a central phosphorylation hub connecting ABA perception with downstream molecular responses	[78,98,101]
ABA-responsive transcriptional regulation	*SnRK2*-dependent activation of stress-responsive transcription factors and associated gene networks	AREB/ABF, NAC, MYB, AP2/ERF	Reprograms expression of ABA- and drought-responsive genes involved in molecular adaptation to water deficit	[99,100]
Post-translational regulation	Ubiquitin-dependent regulation of ABA-associated signaling components	*PeCHYR1*	Adds an additional regulatory layer to ABA signaling and contributes to drought tolerance in *P. euphratica*	[42]
Integrated ABA regulatory network	Coordination among ABA biosynthesis, receptor signaling, phosphorylation, transcriptional regulation, and post-translational control	*NCED*–*PYR*/*PYL*–*PP2C*–*SnRK2*–TF network	Integrates multiple molecular regulatory levels to coordinate drought-responsive signaling in *Populus*	[78,84,85,97]

## Data Availability

No new data were created or analyzed in this study. Data sharing is not applicable to this article.

## References

[1] Allen C.D., Macalady A.K., Chenchouni H., Bachelet D., McDowell N., Vennetier M., Kitzberger T., Rigling A., Breshears D.D., Hogg E.T. (2010). A global overview of drought and heat-induced tree mortality reveals emerging climate change risks for forests. For. Ecol. Manag..

[2] Yohannes H.T., Yu J., Zhang X. (2025). The role of anthropogenic climate change on March–April–May (MAM) drought trends across global land. Int. J. Climatol..

[3] Fang Y., Wang D., Xiao L., Quan M., Qi W., Song F., Zhou J., Liu X., Qin S., Du Q. (2023). Allelic variation in transcription factor PtoWRKY68 contributes to drought tolerance in *Populus*. Plant Physiol..

[4] Han Q., Yang L., Xia L., Zhang H., Zhang S. (2024). Interspecific grafting promotes poplar growth and drought resistance via regulating phytohormone signaling and secondary metabolic pathways. Plant Physiol. Biochem..

[5] Wang D., Jewaria P.K., Xiao J. (2025). Photosynthetic adaptation in poplar under abiotic and biotic stress: Integrating molecular, physiological, and biotechnological perspectives. Int. J. Plant Biol..

[6] Muhammad Aslam M., Waseem M., Jakada B.H., Okal E.J., Lei Z., Saqib H.S.A., Yuan W., Xu W., Zhang Q. (2022). Mechanisms of abscisic acid-mediated drought stress responses in plants. Int. J. Mol. Sci..

[7] Lee S.-U., Mun B.-G., Bae E.-K., Kim J.-Y., Kim H.-H., Shahid M., Choi Y.-I., Hussain A., Yun B.-W. (2021). Drought stress-mediated transcriptome profile reveals NCED as a key player modulating drought tolerance in *Populus davidiana*. Front. Plant Sci..

[8] Wang K., Bu T., Cheng Q., Dong L., Su T., Chen Z., Kong F., Gong Z., Liu B., Li M. (2021). Two homologous LHY pairs negatively control soybean drought tolerance by repressing the abscisic acid responses. New Phytol..

[9] Liu S.J., Zhang H., Jin X.T., Niu M.X., Feng C.H., Liu X., Liu C., Wang H.L., Yin W., Xia X. (2025). PeFUS3 drives lateral root growth via auxin and ABA signalling under drought stress in *Populus*. Plant Cell Environ..

[10] Yang Q., Lee Y. (2021). Two birds with one stone: CEPR2 phosphorylates dual targets in ABA signaling. Mol. Plant.

[11] Zhang X., Liu W., Lv Y., Bai J., Li T., Yang X., Liu L., Zhou H. (2022). Comparative transcriptomics reveals new insights into melatonin-enhanced drought tolerance in naked oat seedlings. PeerJ.

[12] Ali S., Tahir S., Hassan S.S., Lu M., Wang X., Quyen L.T.Q., Zhang W., Chen S. (2025). The role of phytohormones in mediating drought stress responses in *Populus* species. Int. J. Mol. Sci..

[13] Sun J., Xu J., Qu W., Han X., Qiu C., Gai Z., Zhai J., Qin R., Liu H., Wu Z. (2023). Genome-wide analysis of R2R3-MYB transcription factors reveals their differential responses to drought stress and ABA treatment in desert poplar (*Populus euphratica*). Gene.

[14] Song F., Zhou J., Quan M., Xiao L., Lu W., Qin S., Fang Y., Wang D., Li P., Du Q. (2022). Transcriptome and association mapping revealed functional genes respond to drought stress in *Populus*. Front. Plant Sci..

[15] Brunetti C., Gori A., Marino G., Latini P., Sobolev A.P., Nardini A., Haworth M., Giovannelli A., Capitani D., Loreto F. (2019). Dynamic changes in ABA content in water-stressed *Populus nigra*: Effects on carbon fixation and soluble carbohydrates. Ann. Bot..

[16] Wei Y.-S., Javed T., Liu T.-T., Ali A., Gao S.-J. (2025). Mechanisms of abscisic acid (ABA)-mediated plant defense responses: An updated review. Plant Stress.

[17] Chen K., Li G.J., Bressan R.A., Song C.P., Zhu J.K., Zhao Y. (2020). Abscisic acid dynamics, signaling, and functions in plants. J. Integr. Plant Biol..

[18] Robertson S.M., Sakariyahu S.K., Bolaji A., Belmonte M.F., Wilkins O. (2022). Growth-limiting drought stress induces time-of-day-dependent transcriptome and physiological responses in hybrid poplar. AoB Plants.

[19] Cohen D., Bogeat-Triboulot M.-B., Tisserant E., Balzergue S., Martin-Magniette M.-L., Lelandais G., Ningre N., Renou J.-P., Tamby J.-P., Le Thiec D. (2010). Comparative transcriptomics of drought responses in *Populus*: A meta-analysis of genome-wide expression profiling in mature leaves and root apices across two genotypes. BMC Genom..

[20] Huijiao L., Siyue Q., Riqin S., Tongtong Y., Chenxu B., Meijian Y., Yingyue Z., Hong A., Changjun D., Huihui Z. (2026). Physiological and molecular responses of poplar to drought stress and functional analysis of the key factor PagJAZ12B in JA signal transduction to drought tolerance. Tree Physiol..

[21] Wang Y., Yin Z., Li H., Li J., Guo C., Li Z., Zhang H., Wang H., Bai H. (2025). Comprehensive Metabolome and Transcriptome Analysis of *Populus davidiana* and Its Response to Drought Stress. Biology.

[22] Rao S., Tian Y., Zhang C., Qin Y., Liu M., Niu S., Li Y., Chen J. (2023). The JASMONATE ZIM-domain–OPEN STOMATA1 cascade integrates jasmonic acid and abscisic acid signaling to regulate drought tolerance by mediating stomatal closure in poplar. J. Exp. Bot..

[23] Lemaire C., Blackman C.J., Cochard H., Menezes-Silva P.E., Torres-Ruiz J.M., Herbette S. (2021). Acclimation of hydraulic and morphological traits to water deficit delays hydraulic failure during simulated drought in poplar. Tree Physiol..

[24] Muries B., Mom R., Benoit P., Brunel-Michac N., Cochard H., Drevet P., Petel G., Badel E., Fumanal B., Gousset-dupont A. (2019). Aquaporins and water control in drought-stressed poplar leaves: A glimpse into the extraxylem vascular territories. Environ. Exp. Bot..

[25] Guet J., Fichot R., Lédée C., Laurans F., Cochard H., Delzon S., Bastien C., Brignolas F. (2015). Stem xylem resistance to cavitation is related to xylem structure but not to growth and water-use efficiency at the within-population level in *Populus nigra* L.. J. Exp. Bot..

[26] Choat B., Jansen S., Brodribb T.J., Cochard H., Delzon S., Bhaskar R., Bucci S.J., Feild T.S., Gleason S.M., Hacke U.G. (2012). Global convergence in the vulnerability of forests to drought. Nature.

[27] Smith H.K., Puértolas J., Douthe C., Emiliani G., Giovannelli A., Rowland L.S., Allwright M., Bailey-Bale J.H., Valdes-Fragoso P.M., Larsen E.K. (2024). The ideotype for drought tolerance in bioenergy *Populus nigra*. bioRxiv.

[28] Brodribb T.J., Skelton R.P., McAdam S.A., Bienaimé D., Lucani C.J., Marmottant P. (2016). Visual quantification of embolism reveals leaf vulnerability to hydraulic failure. New Phytol..

[29] Jackson M.B., Saker L.R., Crisp C.M., Else M.A., Janowiak F. (2003). Ionic and pH signalling from roots to shoots of flooded tomato plants in relation to stomatal closure. Plant Soil.

[30] Scoffoni C., Jansen S. (2016). I can see clearly now–embolism in leaves. Trends Plant Sci..

[31] Secchi F., Bevilacqua I., Agliassa C., Maghrebi M., Cavalletto S., Morabito C., Lembo S., Vigani G. (2023). Alkaline soil primes the recovery from drought in *Populus nigra* plants through physiological and chemical adjustments. Plant Physiol. Biochem..

[32] Rosso L., Cantamessa S., Bergante S., Biselli C., Fricano A., Chiarabaglio P.M., Gennaro M., Nervo G., Secchi F., Carra A. (2023). Responses to drought stress in poplar: What do we know and what can we learn?. Life.

[33] Khan S.H., Ahmad N., Ahmad F., Kumar R. (2010). Naturally occurring organic osmolytes: From cell physiology to disease prevention. IUBMB Life.

[34] Tschaplinski T.J., Abraham P.E., Jawdy S.S., Gunter L.E., Martin M.Z., Engle N.L., Yang X., Tuskan G.A. (2019). The nature of the progression of drought stress drives differential metabolomic responses in *Populus deltoides*. Ann. Bot..

[35] Guo X.-Y., Zhang X.-S., Huang Z.-Y. (2010). Drought tolerance in three hybrid poplar clones submitted to different watering regimes. J. Plant Ecol..

[36] Gebre G.M., Tschaplinski T.J., Tuskan G.A., Todd D.E. (1998). Clonal and seasonal differences in leaf osmotic potential and organic solutes of five hybrid poplar clones grown under field conditions. Tree Physiol..

[37] Tschaplinski T.J., Tuskan G.A., Gebre G.M., Todd D.E. (1998). Drought resistance of two hybrid *Populus* clones grown in a large-scale plantation. Tree Physiol..

[38] Li X., Yang Y., Sun X., Lin H., Chen J., Ren J., Hu X., Yang Y. (2014). Comparative physiological and proteomic analyses of poplar (*Populus yunnanensis*) plantlets exposed to high temperature and drought. PLoS ONE.

[39] Regier N., Streb S., Cocozza C., Schaub M., Cherubini P., Zeeman S.C., Frey B. (2009). Drought tolerance of two black poplar (*Populus nigra* L.) clones: Contribution of carbohydrates and oxidative stress defence. Plant Cell Environ..

[40] Kapoor D., Singh S., Kumar V., Romero R., Prasad R., Singh J. (2019). Antioxidant enzymes regulation in plants in reference to reactive oxygen species (ROS) and reactive nitrogen species (RNS). Plant Gene.

[41] Yang F., Xu X., Xiao X., Li C. (2009). Responses to drought stress in two poplar species originating from different altitudes. Biol. Plant..

[42] He F., Wang H.L., Li H.G., Su Y., Li S., Yang Y., Feng C.H., Yin W., Xia X. (2018). Pe CHYR 1, a ubiquitin E3 ligase from *Populus euphratica*, enhances drought tolerance via ABA-induced stomatal closure by ROS production in *Populus*. Plant Biotechnol. J..

[43] Yang Y., Li H.-G., Wang J., Wang H.-L., He F., Su Y., Zhang Y., Feng C.-H., Niu M., Li Z. (2020). ABF3 enhances drought tolerance via promoting ABA-induced stomatal closure by directly regulating ADF5 in *Populus euphratica*. J. Exp. Bot..

[44] Ye Z.-Q., Wang J.-M., Wang W.-J., Zhang T.-H., Li J.-W. (2019). Effects of root phenotypic changes on the deep rooting of *Populus euphratica* seedlings under drought stresses. PeerJ.

[45] Paul S., Wildhagen H., Janz D., Polle A. (2018). Drought effects on the tissue-and cell-specific cytokinin activity in poplar. AoB Plants.

[46] Sun Z., Shen Y., Niinemets Ü. (2020). Responses of isoprene emission and photochemical efficiency to severe drought combined with prolonged hot weather in hybrid *Populus*. J. Exp. Bot..

[47] Yang Y., Zhao J., Ren X., Bai X., Li T., Li J. (2025). PsDUF6A from *Populus simonii* enhances drought tolerance in transgenic *Arabidopsis* and poplar by increasing ROS scavenging. Plant Stress.

[48] Khan N. (2025). Molecular insights into ABA-mediated regulation of stress tolerance and development in plants. Int. J. Mol. Sci..

[49] Giordano M., Petropoulos S.A., Rouphael Y. (2021). Response and defence mechanisms of vegetable crops against drought, heat and salinity stress. Agriculture.

[50] Perreau F., Frey A., Effroy-Cuzzi D., Savane P., Berger A., Gissot L., Marion-Poll A. (2020). ABSCISIC ACID-DEFICIENT4 has an essential function in both cis-violaxanthin and cis-neoxanthin synthesis. Plant Physiol..

[51] Seo M., Marion-Poll A. (2019). Abscisic acid metabolism and transport. Advances in Botanical Research.

[52] Vishwakarma K., Upadhyay N., Kumar N., Yadav G., Singh J., Mishra R.K., Kumar V., Verma R., Upadhyay R., Pandey M. (2017). Abscisic acid signaling and abiotic stress tolerance in plants: A review on current knowledge and future prospects. Front. Plant Sci..

[53] Qin X., Zeevaart J.A. (1999). The 9-cis-epoxycarotenoid cleavage reaction is the key regulatory step of abscisic acid biosynthesis in water-stressed bean. Proc. Natl. Acad. Sci. USA.

[54] Thompson A.J., Jackson A.C., Parker R.A., Morpeth D.R., Burbidge A., Taylor I.B. (2000). Abscisic acid biosynthesis in tomato: Regulation of zeaxanthin epoxidase and 9-cis-epoxycarotenoid dioxygenase mRNAs by light/dark cycles, water stress and abscisic acid. Plant Mol. Biol..

[55] Mun B.-G., Hussain A., Park E.-J., Lee S.-U., Sharma A., Imran Q.M., Jung K.-H., Yun B.-W. (2017). Profile and time-scale dynamics of differentially expressed genes in transcriptome of *Populus davidiana* under drought stress. Plant Mol. Biol. Report..

[56] Zhang X., Zang R., Li C. (2004). Population differences in physiological and morphological adaptations of *Populus davidiana* seedlings in response to progressive drought stress. Plant Sci..

[57] Popko J., Hänsch R., Mendel R.R., Polle A., Teichmann T. (2010). The role of abscisic acid and auxin in the response of poplar to abiotic stress. Plant Biol..

[58] Bian Z., Wang D., Liu Y., Xi Y., Wang X., Meng S. (2021). Analysis of *Populus* glycosyl hydrolase family I members and their potential role in the ABA treatment and drought stress response. Plant Physiol. Biochem..

[59] Tan B.C., Joseph L.M., Deng W.T., Liu L., Li Q.B., Cline K., McCarty D.R. (2003). Molecular characterization of the *Arabidopsis* 9-cis epoxycarotenoid dioxygenase gene family. Plant J..

[60] González-Guzmán M., Apostolova N., Bellés J.M., Barrero J.M., Piqueras P., Ponce M.R., Micol J.L., Serrano R., Rodríguez P.L. (2002). The short-chain alcohol dehydrogenase ABA2 catalyzes the conversion of xanthoxin to abscisic aldehyde. Plant Cell.

[61] Seo M., Peeters A.J., Koiwai H., Oritani T., Marion-Poll A., Zeevaart J.A., Koornneef M., Kamiya Y., Koshiba T. (2000). The *Arabidopsis* aldehyde oxidase 3 (AAO3) gene product catalyzes the final step in abscisic acid biosynthesis in leaves. Proc. Natl. Acad. Sci. USA.

[62] Endo A., Koshiba T., Kamiya Y., Nambara E. (2008). Vascular system is a node of systemic stress responses: Competence of the cell to synthesize abscisic acid and its responsiveness to external cues. Plant Signal. Behav..

[63] Kuromori T., Miyaji T., Yabuuchi H., Shimizu H., Sugimoto E., Kamiya A., Moriyama Y., Shinozaki K. (2010). ABC transporter AtABCG25 is involved in abscisic acid transport and responses. Proc. Natl. Acad. Sci. USA.

[64] Huang X., Zhang X., An N., Zhang M., Ma M., Yang Y., Jing L., Wang Y., Chen Z., Zhang P. (2023). Cryo-EM structure and molecular mechanism of abscisic acid transporter ABCG25. Nat. Plants.

[65] Kang J., Hwang J.-U., Lee M., Kim Y.-Y., Assmann S.M., Martinoia E., Lee Y. (2010). PDR-type ABC transporter mediates cellular uptake of the phytohormone abscisic acid. Proc. Natl. Acad. Sci. USA.

[66] Kanno Y., Hanada A., Chiba Y., Ichikawa T., Nakazawa M., Matsui M., Koshiba T., Kamiya Y., Seo M. (2012). Identification of an abscisic acid transporter by functional screening using the receptor complex as a sensor. Proc. Natl. Acad. Sci. USA.

[67] Bai H., Euring D., Volmer K., Janz D., Polle A. (2013). The nitrate transporter (NRT) gene family in poplar. PLoS ONE.

[68] Chen S., Wang S. (1997). Genotypic variation in drought tolerance of poplar in relation to abscisic acid. Tree Physiol..

[69] Rehaman A., Khan S., Rawat B., Gaira K.S., Asgher M., Semwal P., Tripathi V. (2025). Mechanistic insights into plant drought tolerance: A multi-level perspective. J. Crop Health.

[70] Kuromori T., Seo M., Shinozaki K. (2018). ABA transport and plant water stress responses. Trends Plant Sci..

[71] Hartung W., Sauter A., Hose E. (2002). Abscisic acid in the xylem: Where does it come from, where does it go to?. J. Exp. Bot..

[72] Kocurek M., Gieniec M., Waligórski P., Miszalski Z. (2025). Stem Photosynthesis in ‘Hybrid Poplar 275’ Remains Stable Following Defoliation Induced by Severe Drought. Forests.

[73] Mo W., Zheng X., Shi Q., Zhao X., Chen X., Yang Z., Zuo Z. (2024). Unveiling the crucial roles of abscisic acid in plant physiology: Implications for enhancing stress tolerance and productivity. Front. Plant Sci..

[74] Jhanji S., Goyal E., Chumber M., Kaur G. (2024). Exploring fine tuning between phytohormones and ROS signaling cascade in regulation of seed dormancy, germination and seedling development. Plant Physiol. Biochem..

[75] Jakubowicz M., Nowak W., Gałgański Ł., Babula-Skowrońska D. (2018). Expression profiling of genes encoding ABA route components in response to dehydration or various light conditions in poplar buds and leaves. J. Plant Physiol..

[76] Li Q., Shen C., Zhang Y., Zhou Y., Niu M., Wang H.-L., Lian C., Tian Q., Mao W., Wang X. (2023). PePYL4 enhances drought tolerance by modulating water-use efficiency and ROS scavenging in *Populus*. Tree Physiol..

[77] Li J., Jia H., Zhang J., Sun J., Zhang Y., Lu M., Xin X., Hu J. (2018). Genome-wide characterization of protein phosphatase 2C genes in *Populus euphratica* and their expression profiling under multiple abiotic stresses. Tree Genet. Genomes.

[78] Yu X., Takebayashi A., Demura T., Ohtani M. (2017). Differential expression of poplar sucrose nonfermenting1-related protein kinase 2 genes in response to abiotic stress and abscisic acid. J. Plant Res..

[79] Jin H., Li J., Song T., Miao D., Ning Q., Zhang X., Gai Z., Li Z., Jiao P., Wu Z. (2025). Genome-wide identification of the SnRK2 gene family and its response to abiotic stress in *Populus euphratica*. Int. J. Mol. Sci..

[80] Zha D., He Y., Song J. (2025). Regulatory role of ABA-responsive element binding factors in plant abiotic stress response. Physiol. Plant..

[81] Li S., Lin Y.-C.J., Wang P., Zhang B., Li M., Chen S., Shi R., Tunlaya-Anukit S., Liu X., Wang Z. (2019). The AREB1 transcription factor influences histone acetylation to regulate drought responses and tolerance in *Populus trichocarpa*. Plant Cell.

[82] Liu H., Song S., Zhang H., Li Y., Niu L., Zhang J., Wang W. (2022). Signaling transduction of ABA, ROS, and Ca^2+^ in plant stomatal closure in response to drought. Int. J. Mol. Sci..

[83] Kaya C., Uğurlar F., Adamakis I.-D.S. (2024). Molecular mechanisms of CBL-CIPK signaling pathway in plant abiotic stress tolerance and hormone crosstalk. Int. J. Mol. Sci..

[84] Cutler S.R., Rodriguez P.L., Finkelstein R.R., Abrams S.R. (2010). Abscisic acid: Emergence of a core signaling network. Annu. Rev. Plant Biol..

[85] Fujii H., Chinnusamy V., Rodrigues A., Rubio S., Antoni R., Park S.-Y., Cutler S.R., Sheen J., Rodriguez P.L., Zhu J.-K. (2009). In vitro reconstitution of an abscisic acid signalling pathway. Nature.

[86] Nambara E., Marion-Poll A. (2005). Abscisic acid biosynthesis and catabolism. Annu. Rev. Plant Biol..

[87] Fidler J., Graska J., Gietler M., Nykiel M., Prabucka B., Rybarczyk-Płońska A., Muszyńska E., Morkunas I., Labudda M. (2022). PYR/PYL/RCAR receptors play a vital role in the abscisic-acid-dependent responses of plants to external or internal stimuli. Cells.

[88] Yu J., Yang L., Liu X., Tang R., Wang Y., Ge H., Wu M., Zhang J., Zhao F., Luan S. (2016). Overexpression of poplar pyrabactin resistance-like abscisic acid receptors promotes abscisic acid sensitivity and drought resistance in transgenic *Arabidopsis*. PLoS ONE.

[89] Zhao Y., Chan Z., Gao J., Xing L., Cao M., Yu C., Hu Y., You J., Shi H., Zhu Y. (2016). ABA receptor PYL9 promotes drought resistance and leaf senescence. Proc. Natl. Acad. Sci. USA.

[90] Li Q., Tian Q., Zhang Y., Niu M., Yu X., Lian C., Liu C., Wang H.-L., Yin W., Xia X. (2022). Increased abscisic acid sensitivity and drought tolerance of *Arabidopsis* by overexpression of poplar abscisic acid receptors. Plant Cell Tissue Organ Cult. (PCTOC).

[91] Zhang J., Peng M., Chen P., Yao S., He Y., Wang D., Agassin R.H., Ji K. (2024). PmbHLH58 from *Pinus massoniana* Improves Drought Tolerance by Reducing Stomatal Aperture and Inducing ABA Receptor Family Genes in Transgenic Poplar Plants. Int. J. Mol. Sci..

[92] Yu J., Ge H., Wang X., Tang R., Wang Y., Zhao F., Lan W., Luan S., Yang L. (2017). Overexpression of pyrabactin resistance-like abscisic acid receptors enhances drought, osmotic, and cold tolerance in transgenic poplars. Front. Plant Sci..

[93] Takase H., Saigusa M., Yamashita K., Umezawa T. (2026). Dynamic and Basal Phosphorylation Landscapes of Abscisic Acid Signaling Revealed by Phosphoproteome Analysis in *Arabidopsis*. Int. J. Mol. Sci..

[94] Umezawa T., Nakashima K., Miyakawa T., Kuromori T., Tanokura M., Shinozaki K., Yamaguchi-Shinozaki K. (2010). Molecular basis of the core regulatory network in ABA responses: Sensing, signaling and transport. Plant Cell Physiol..

[95] He F., Niu M.-X., Wang T., Li J.-L., Shi Y.-J., Zhao J.-J., Li H., Xiang X., Yang P., Wei S.-Y. (2024). The ubiquitin E3 ligase RZFP1 affects drought tolerance in poplar by mediating the degradation of the protein phosphatase PP2C-9. Plant Physiol..

[96] Ghanizadeh H., Qamer Z., Zhang Y., Wang A. (2025). The multifaceted roles of PP2C phosphatases in plant growth, signaling, and response to abiotic and biotic stress. Plant Commun..

[97] Papacek M., Christmann A., Grill E. (2017). Interaction network of ABA receptors in grey poplar. Plant J..

[98] Wang P., Xue L., Batelli G., Lee S., Hou Y.-J., Van Oosten M.J., Zhang H., Tao W.A., Zhu J.-K. (2013). Quantitative phosphoproteomics identifies SnRK2 protein kinase substrates and reveals the effectors of abscisic acid action. Proc. Natl. Acad. Sci. USA.

[99] Zhang H., Wang M., Xing X., Zeng D., Liu X., Ren Z., Yu S., Liu H., Yu S., Zhou C. (2026). Integrated physiological and transcriptomic analysis revealed key genes and pathways related to continuous drought and salinity stress in *Populus*. Plant Cell Rep..

[100] Liu T., Luo T., Guo X., Zou X., Zhou D., Afrin S., Li G., Zhang Y., Zhang R., Luo Z. (2019). PgMYB2, a MeJA-responsive transcription factor, positively regulates the dammarenediol synthase gene expression in *Panax ginseng*. Int. J. Mol. Sci..

[101] Soma F., Takahashi F., Yamaguchi-Shinozaki K., Shinozaki K. (2021). Cellular phosphorylation signaling and gene expression in drought stress responses: ABA-dependent and ABA-independent regulatory systems. Plants.

[102] Zou J., Sun J., Liu H., Li B., Zhu T., Jin C., Li X., Jin H. (2023). Genes and pathways associated with drought tolerance in *Populus wutunensis* under drought stress. Res. Sq..

[103] Ahkami A.H. (2023). Systems biology of root development in *Populus*: Review and perspectives. Plant Sci..

[104] Salvi P., Manna M., Kaur H., Thakur T., Gandass N., Bhatt D., Muthamilarasan M. (2021). Phytohormone signaling and crosstalk in regulating drought stress response in plants. Plant Cell Rep..

[105] Musazade E., Mrisho I.I., Feng X. (2025). Auxin metabolism and signaling: Integrating independent mechanisms and crosstalk in plant abiotic stress responses. Plant Stress.

[106] Zhou Y., Zhang Y., Wang X., Han X., An Y., Lin S., Shen C., Wen J., Liu C., Yin W. (2020). Root-specific NF-Y family transcription factor, PdNF-YB21, positively regulates root growth and drought resistance by abscisic acid-mediated indoylacetic acid transport in *Populus*. New Phytol..

[107] Roychowdhury R., Hada A., Biswas S., Mishra S., Prusty M.R., Das S.P., Ray S., Kumar A., Sarker U. (2025). Jasmonic acid (JA) in plant immune response: Unravelling complex molecular mechanisms and networking of defence signalling against pathogens. J. Plant Growth Regul..

[108] Liu X.D., Zeng Y.Y., Hasan M.M., Ghimire S., Jiang H., Qi S.H., Tian X.Q., Fang X.W. (2024). Diverse functional interactions between ABA and ethylene in plant development and responses to stress. Physiol. Plant..

[109] Marino G., Brunetti C., Tattini M., Romano A., Biasioli F., Tognetti R., Loreto F., Ferrini F., Centritto M. (2017). Dissecting the role of isoprene and stress-related hormones (ABA and ethylene) in *Populus nigra* exposed to unequal root zone water stress. Tree Physiol..

[110] Huan X., Wang X., Zou S., Zhao K., Han Y., Wang S. (2023). Transcription factor ERF194 modulates the stress-related physiology to enhance drought tolerance of poplar. Int. J. Mol. Sci..

[111] Sun L., Dong X., Song X. (2023). PtrABR1 increases tolerance to drought stress by enhancing lateral root formation in *Populus trichocarpa*. Int. J. Mol. Sci..

[112] Nishiyama R., Watanabe Y., Fujita Y., Le D.T., Kojima M., Werner T., Vankova R., Yamaguchi-Shinozaki K., Shinozaki K., Kakimoto T. (2011). Analysis of cytokinin mutants and regulation of cytokinin metabolic genes reveals important regulatory roles of cytokinins in drought, salt and abscisic acid responses, and abscisic acid biosynthesis. Plant Cell.

[113] Zwack P.J., Rashotte A.M. (2015). Interactions between cytokinin signalling and abiotic stress responses. J. Exp. Bot..

[114] Cortleven A., Leuendorf J.E., Frank M., Pezzetta D., Bolt S., Schmülling T. (2019). Cytokinin action in response to abiotic and biotic stresses in plants. Plant Cell Environ..

[115] Li M., Zhou S., Jiang M., Geng J., Ni H., Li G., Zhao Y., Dong Y. (2026). ABA–cytokinin crosstalk regulate drought tolerance in *Ziziphus jujuba* var. spinosa through the phenylpropanoid pathway: Insights from physiological and multi-omics integration. Plant Physiol. Biochem..

[116] Yu T., Ma X., Zhang J., Cao S., Li W., Yang G., He C. (2025). Progress in transcriptomics and metabolomics in plant responses to abiotic stresses. Curr. Issues Mol. Biol..

[117] Huang H., Ullah F., Zhou D.-X., Yi M., Zhao Y. (2019). Mechanisms of ROS regulation of plant development and stress responses. Front. Plant Sci..

[118] Colebrook E.H., Thomas S.G., Phillips A.L., Hedden P. (2014). The role of gibberellin signalling in plant responses to abiotic stress. J. Exp. Biol..

[119] Achard P., Cheng H., De Grauwe L., Decat J., Schoutteten H., Moritz T., Van Der Straeten D., Peng J., Harberd N.P. (2006). Integration of plant responses to environmentally activated phytohormonal signals. Science.

[120] Yu D., Wildhagen H., Tylewicz S., Miskolczi P.C., Bhalerao R.P., Polle A. (2019). Abscisic acid signalling mediates biomass trade-off and allocation in poplar. New Phytol..

[121] Kim T.-L., Lim H., Denison M.I.J., Oh C. (2023). Transcriptomic and physiological analysis reveals genes associated with drought stress responses in *Populus alba* × *Populus glandulosa*. Plants.

[122] Byeon S., Lee I.H., Kim T.-L., Jang H.-A. (2024). Comparative transcriptomic analysis reveals the underlying molecular mechanisms of drought tolerance in *Populus davidiana* and its hybrid with *P. alba*. Plant Biotechnol. Rep..

[123] Kapazoglou A., Tani E., Papasotiropoulos V., Letsiou S., Gerakari M., Abraham E., Bebeli P.J. (2025). Enhancing abiotic stress resilience in mediterranean woody perennial fruit crops: Genetic, epigenetic, and microbial molecular perspectives in the face of climate change. Int. J. Mol. Sci..

[124] Joshi R., Wani S.H., Singh B., Bohra A., Dar Z.A., Lone A.A., Pareek A., Singla-Pareek S.L. (2016). Transcription factors and plants response to drought stress: Current understanding and future directions. Front. Plant Sci..

[125] Bogeat-Triboulot M.-B., Brosché M., Renaut J., Jouve L., Le Thiec D., Fayyaz P., Vinocur B., Witters E., Laukens K., Teichmann T. (2007). Gradual soil water depletion results in reversible changes of gene expression, protein profiles, ecophysiology, and growth performance in *Populus euphratica*, a poplar growing in arid regions. Plant Physiol..

[126] Hamanishi E.T., Barchet G.L., Dauwe R., Mansfield S.D., Campbell M.M. (2015). Poplar trees reconfigure the transcriptome and metabolome in response to drought in a genotype-and time-of-day-dependent manner. BMC Genom..

[127] Li G., Feng B., Zhu Q.-H., Jiang K., Zhang T. (2025). Protein post-translational modifications in plant abiotic stress responses. Plants.

[128] Seo J., Lee K.-J. (2004). Post-translational modifications and their biological functions: Proteomic analysis and systematic approaches. J. Biochem. Mol. Biol..

[129] Chen J., Zhang D., Zhang C., Xia X., Yin W., Tian Q. (2015). A putative PP2C-encoding gene negatively regulates ABA signaling in *Populus euphratica*. PLoS ONE.

[130] Jia H., Wang L., Li J., Sun P., Lu M., Hu J. (2020). Comparative metabolomics analysis reveals different metabolic responses to drought in tolerant and susceptible poplar species. Physiol. Plant..

[131] Duan C.-G., Zhu J.-K., Cao X. (2018). Retrospective and perspective of plant epigenetics in China. J. Genet. Genom..

[132] Chang Y.N., Zhu C., Jiang J., Zhang H., Zhu J.K., Duan C.G. (2020). Epigenetic regulation in plant abiotic stress responses. J. Integr. Plant Biol..

[133] Zhang B., Wang Z., Dai X., Gao J., Zhao J., Ma R., Chen Y., Sun Y., Ma H., Li S. (2024). A COMPASS histone H3K4 trimethyltransferase pentamer transactivates drought tolerance and growth/biomass production in *Populus trichocarpa*. New Phytol..

[134] Jiang J., Ding A.B., Liu F., Zhong X. (2020). Linking signaling pathways to histone acetylation dynamics in plants. J. Exp. Bot..

[135] Duan H., Lu X., Lian C., An Y., Xia X., Yin W. (2016). Genome-wide analysis of microRNA responses to the phytohormone abscisic acid in *Populus euphratica*. Front. Plant Sci..

[136] Bloch D., Puli M.R., Mosquna A., Yalovsky S. (2019). Abiotic stress modulates root patterning via ABA-regulated microRNA expression in the endodermis initials. Development.

[137] Zhang L., Liu J., Cheng J., Sun Q., Zhang Y., Liu J., Li H., Zhang Z., Wang P., Cai C. (2022). LncRNA7 and lncRNA2 modulate cell wall defense genes to regulate cotton resistance to Verticillium wilt. Plant Physiol..

[138] Ding Y., Fromm M., Avramova Z. (2012). Multiple exposures to drought’train’transcriptional responses in *Arabidopsis*. Nat. Commun..

[139] Liu N., Fromm M., Avramova Z. (2014). H3K27me3 and H3K4me3 chromatin environment at super-induced dehydration stress memory genes of *Arabidopsis thaliana*. Mol. Plant.

[140] Seki M., Narusaka M., Abe H., Kasuga M., Yamaguchi-Shinozaki K., Carninci P., Hayashizaki Y., Shinozaki K. (2001). Monitoring the expression pattern of 1300 *Arabidopsis* genes under drought and cold stresses by using a full-length cDNA microarray. Plant Cell.

[141] Kumar K., Banerjee S., Malik A., Tiwari N., Bhusal P., Kalotra N.K., Singh H., Barthwal S. (2026). Foliar application of glutamic acid enhances drought tolerance in poplar by coordinating physiological, molecular, and phyllosphere microbial responses. Discov. Plants.

[142] Liang D., Zhang Z., Wu H., Huang C., Shuai P., Ye C.-Y., Tang S., Wang Y., Yang L., Wang J. (2014). Single-base-resolution methylomes of *Populus trichocarpa* reveal the association between DNA methylation and drought stress. BMC Genet..

[143] González R.M., Ricardi M.M., Iusem N.D. (2013). Epigenetic marks in an adaptive water stress-responsive gene in tomato roots under normal and drought conditions. Epigenetics.

[144] Li B., Qin Y., Duan H., Yin W., Xia X. (2011). Genome-wide characterization of new and drought stress responsive microRNAs in *Populus euphratica*. J. Exp. Bot..

[145] Zhang T., Zhang W., Ding C., Hu Z., Fan C., Zhang J., Li Z., Diao S., Shen L., Zhang B. (2023). A breeding strategy for improving drought and salt tolerance of poplar based on CRISPR/Cas9. Plant Biotechnol. J..

[146] Vinocur B., Altman A. (2005). Recent advances in engineering plant tolerance to abiotic stress: Achievements and limitations. Curr. Opin. Biotechnol..

[147] Zhu J.-K. (2016). Abiotic stress signaling and responses in plants. Cell.

[148] Baulcombe D. (2004). RNA silencing in plants. Nature.

[149] Geng L., Ren J., Ji X., Yan S., Song X.S. (2023). Over-expression of DREB46 enhances drought tolerance in *Populus trichocarpa*. J. Plant Physiol..

[150] Kaya C., Uğurlar F., Adamakis I.-D.S. (2024). Epigenetic modifications of hormonal signaling pathways in plant drought response and tolerance for sustainable food security. Int. J. Mol. Sci..

[151] Zuo Z.-F., Kang H.-G., Hong Q.-C., Park M.-Y., Sun H.-J., Kim J., Song P.-S., Lee H.-Y. (2020). A novel basic helix-loop-helix transcription factor, ZjICE2 from *Zoysia japonica* confers abiotic stress tolerance to transgenic plants via activating the DREB/CBF regulon and enhancing ROS scavenging. Plant Mol. Biol..

[152] Erdoğan İ., Cevher-Keskin B., Bilir Ö., Hong Y., Tör M. (2023). Recent developments in CRISPR/Cas9 genome-editing technology related to plant disease resistance and abiotic stress tolerance. Biology.

[153] Joshi A., Yang S.-Y., Song H.-G., Min J., Lee J.-H. (2023). Genetic databases and gene editing tools for enhancing crop resistance against abiotic stress. Biology.

[154] Santosh Kumar V., Verma R.K., Yadav S.K., Yadav P., Watts A., Rao M., Chinnusamy V. (2020). CRISPR-Cas9 mediated genome editing of drought and salt tolerance (OsDST) gene in indica mega rice cultivar MTU1010. Physiol. Mol. Biol. Plants.

[155] Shi J., Gao H., Wang H., Lafitte H.R., Archibald R.L., Yang M., Hakimi S.M., Mo H., Habben J.E. (2017). ARGOS 8 variants generated by CRISPR-Cas9 improve maize grain yield under field drought stress conditions. Plant Biotechnol. J..

[156] Liu J., Ye M., Fan Z., Wang Y., Chen J., Li H., Deng X., Kim H.S., Kwak S.-S., Ke Q. (2025). PagHCF106 negatively regulates drought stress tolerance in poplar (*Populus alba* × *Populus glandulosa*) by modulating stomatal aperture. Tree Physiol..

[157] Pak S., Li C. (2022). Progress and challenges in applying CRISPR/Cas techniques to the genome editing of trees. For. Res..

[158] Cao H.X., Vu G.T.H., Gailing O. (2022). From genome sequencing to CRISPR-based genome editing for climate-resilient forest trees. Int. J. Mol. Sci..

[159] Osakabe Y., Osakabe K., Shinozaki K., Tran L.-S.P. (2014). Response of plants to water stress. Front. Plant Sci..

[160] Ma X., Zhang Q., Zhu Q., Liu W., Chen Y., Qiu R., Wang B., Yang Z., Li H., Lin Y. (2015). A robust CRISPR/Cas9 system for convenient, high-efficiency multiplex genome editing in monocot and dicot plants. Mol. Plant.

[161] Yoshida T., Mogami J., Yamaguchi-Shinozaki K. (2014). ABA-dependent and ABA-independent signaling in response to osmotic stress in plants. Curr. Opin. Plant Biol..

[162] Gao C. (2021). Genome engineering for crop improvement and future agriculture. Cell.

[163] Boyne A., Yang M., Pulicani S., Feola M., Tkach D., Hong R., Duclert A., Duchateau P., Juillerat A. (2022). Efficient multitool/multiplex gene engineering with TALE-BE. Front. Bioeng. Biotechnol..

[164] Lowder L.G., Zhang D., Baltes N.J., Paul J.W., Tang X., Zheng X., Voytas D.F., Hsieh T.-F., Zhang Y., Qi Y. (2015). A CRISPR/Cas9 toolbox for multiplexed plant genome editing and transcriptional regulation. Plant Physiol..

[165] Piatek A., Ali Z., Baazim H., Li L., Abulfaraj A., Al-Shareef S., Aouida M., Mahfouz M.M. (2015). RNA-guided transcriptional regulation in planta via synthetic dC as9-based transcription factors. Plant Biotechnol. J..

[166] Papikian A., Liu W., Gallego-Bartolomé J., Jacobsen S.E. (2019). Site-specific manipulation of Arabidopsis loci using CRISPR-Cas9 SunTag systems. Nat. Commun..

[167] Goralogia G.S., Redick T.P., Strauss S.H. (2021). Gene editing in tree and clonal crops: Progress and challenges. Vitro Cell. Dev. Biol.-Plant.

